# Incorporation of Al_2_O_3_, GO, and Al_2_O_3_@GO nanoparticles into water-borne epoxy coatings: abrasion and corrosion resistance†

**DOI:** 10.1039/d2ra04223a

**Published:** 2022-08-31

**Authors:** Jia-qi Huang, Kunming Liu, Xinlong Song, Guocheng Zheng, Qing Chen, Jiadi Sun, Haozhe Jin, Lanlan Jiang, Yusheng Jiang, Yi Zhang, Peng Jiang, Wangping Wu

**Affiliations:** Electrochemistry and Corrosion Laboratory, School of Mechanical Engineering and Rail Transit, Changzhou University Changzhou 213164 China wwp3.14@163.com wuwping@cczu.edu.cn; Jiangsu Kexiang Anticorrosion Materials Co., Ltd Changzhou 213100 China; Zhejiang Fangyuan Test Group Hangzhou 310018 China; CNOOC Changzhou Paint and Coatings Industry Research Institute Co., Ltd Changzhou 213016 China; Key Lab. of Fluid Transmission Technology of Zhejiang Province, Zhejiang, Sci-Tech University Hangzhou, 310018 China

## Abstract

Nano-Al_2_O_3_ particles and graphene oxide (GO) nanosheets were modified by 3-aminopropyltriethoxysilane (KH550), and then dispersed in epoxy resin, and finally modified-Al_2_O_3_/epoxy, modified-GO/epoxy and modified-Al_2_O_3_@GO/epoxy composite coatings were prepared on steel sheets by the scraping stick method. The microstructure, phase identification, surface bonding and composition of the nanoparticles were characterized by SEM, XRD, FT-IR, and Raman spectroscopy, respectively. The hardness of the coating was assessed by the pencil hardness method. The abrasion resistance of the coating was tested by a sand washing machine. The corrosion resistance of the coating was assessed using salt spray, a long-period immersion test, potentiodynamic polarization curves and electrochemical impedance spectra. With the addition of a small amount of nanoparticles, the dispersion of nanoparticles in the epoxy resin was good. When the content of nano-Al_2_O_3_ particles was equal to 1.5 wt%, the particles in the epoxy exhibited the best dispersion and stability. However, the GO and Al_2_O_3_@GO nanofillers in the epoxy resin exhibited poor dispersion and stability. The hardness, abrasion and corrosion resistance of the composite coatings were improved with the addition of a small amount of nanoparticles, but the performance began to decline after exceeding a certain content range of the nanoparticles. A relatively good abrasion resistance for the coatings was obtained when the content of Al_2_O_3_, GO and Al_2_O_3_@GO after modification was 1.5 wt%, 0.2 wt% and 0.4 wt%, respectively. The corrosion resistance of the coatings doped with nano-Al_2_O_3_ particles was better than that of the coatings incorporating GO nanosheets and Al_2_O_3_@GO hybrids. The corrosion mechanism of the composite coatings in 3.5 wt% NaCl solution was addressed and studied.

## Abstract 2

Nano-Al_2_O_3_ particles and graphene oxide (GO) nanosheets were modified by 3-aminopropyltriethoxysilane (KH550), and then dispersed in epoxy resin, and finally modified-Al_2_O_3_/epoxy, modified-GO/epoxy and modified-Al_2_O_3_@GO/epoxy composite coatings were prepared on steel sheets by the scraping stick method.

## Graphical abstract

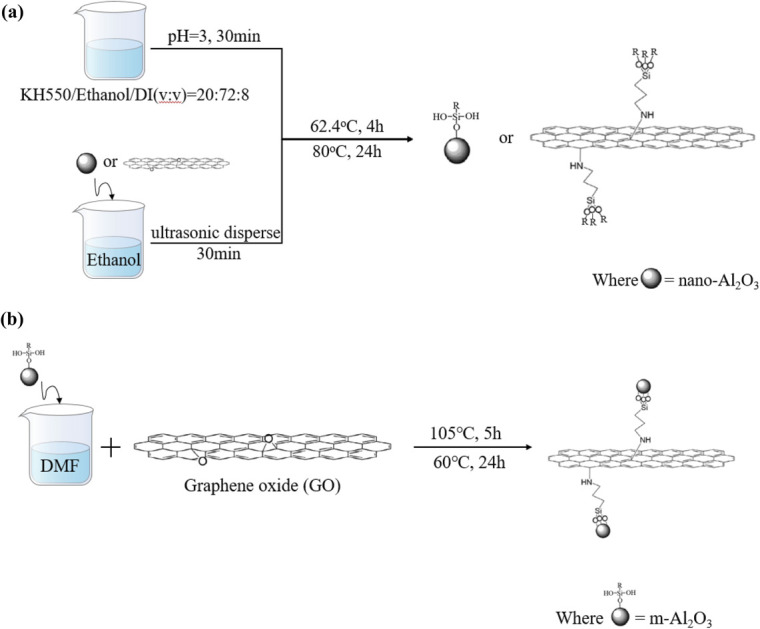

## 1 Introduction

In the field of anticorrosion, water-borne epoxy resin plays an important role due to its excellent mechanical and chemical properties.^1^ However, the protective performance of epoxy resin coating still has some room for improvement. Microcracks and holes are generated in the epoxy coating because of the influence of the high crosslinking density of the network. Corrosion media such as H_2_O, O_2_, and Cl^−^ ions will permeate the epoxy coatings through some minor defects, which will reduce the service term of the coatings in the corrosive environment. Nanoparticles provide a new approach for modifying epoxy resin to obtain good mechanical properties and abrasion and corrosion resistance of the epoxy coatings. Nanoparticles have many unique and excellent properties. The insulation oxide ceramic nanoparticles, such as TiO_2_, SiO_2_, Al_2_O_3_, and ZrO_2_, acted as fillers for epoxy resin, which can improve the properties of the epoxy resin. Among the numerous oxide ceramic nanoparticles, the low-cost nano-alumina (nano-Al_2_O_3_) is well-known for its excellent mechanical properties, high thermal stability, super electrical insulation properties, and high surface area, which can be acted as a candidate reinforcement filler.^2^ However, due to the small size and high specific surface area, nano-Al_2_O_3_ particles are easy to agglomerate, resulting in its poor dispersion, which could influence the properties and performance of the composite coatings.^3^ Salimi et al.^4^ prepared Al_2_O_3_/tetraglycidyl-4,4′-diaminodiphenylmethane nanocomposites by adding nano-Al_2_O_3_ particles modified by aminopropyltriethoxysilaneandglycidylpropyltrimethoxysilane into the epoxy. The dispersion of nanoparticles in the resin influenced curing exothermic and fracture morphology of the composites, the dispersibility of nanoparticles modified by silane was good.^5^ The mechanical properties and corrosion resistance of the epoxy coatings can be significantly enhanced with the addition of nano-Al_2_O_3_.^6–8^

Graphene oxide (GO) has a two-dimensional lamellar structure and large specific surface area, which contains many active functional groups, such as hydroxyl group (–OH), carboxyl group (–COOH), epoxy group (–CH(O)CH–).^9^ These functional groups make GO have good wetting and high surface activity, and can improve the compatibility between GO and epoxy resin^10^. Recently, the corrosion and tribology properties of graphene-based epoxy coating have attracted tremendous attention, due to its super physical shielding and high lubricity.^11–13^ Cui *et al.*^14^ reported an eco-friendly water-borne epoxy coating by embedding GO nanosheets, and found that inclusion of well-dispersed GO-polymerized polydopamine nanosheets led to the remarkable improvement in the corrosion resistance of water-borne epoxy coating. However, during the preparation process for the coatings, the dispersibility of GO nanosheets is one of the key factors due to the agglomeration of GO nanosheets with their high specific area, and nanoscale internal van der Waals forces, which limits the improvement in the abrasion and corrosion resistance of the epoxy coatings.^15,16^ The charged GO nanosheets^17^ or a new fangled cationic dopamine-reduced GO^18^ nanosheets that can stably disperse in the water-based epoxy. The highly parallel GO nanosheets tremendously improve the physical barrier effect of the coatings and prolong the penetration path of the corrosive medium. Li *et al.*^19^ added the epoxy resin into the ethanol solution containing sodium polystyrene sulfonate modified GO by the ultrasonic, stirring and volatile solvent methods to realize the uniform filling of GO. Pathak *et al.*^20^ used the phase transfer method to realize the uniform dispersion of GO in epoxy resin. Li *et al.*^21^ studied the properties of silane-coupling-agent-modified GO composite epoxy coatings, and showed that modified GO remarkably enhanced the mechanical properties of epoxy coatings.

Graphene oxide–alumina (GO–Al_2_O_3_) hybrids have the physical barrier effect of GO and reinforcement of nano-Al_2_O_3_, resulting a hybrid property of the improvement in the mechanical properties-corrosion resistance of the composite coatings.^22^ Nano-Al_2_O_3_ particles are acted as a secondary filler to isolate the GO nanosheets. At the same time, nano-Al_2_O_3_ particles can weaken the van der Waals force of GO nanosheets and improve the dispersibility of GO nanosheets in epoxy resin^23^. Osman *et al.*^24^ found that the settling of nano-Al_2_O_3_ particles on the graphene surface not only inhibited the electron transfer but also eliminated the agglomerations of graphene. However, the excess addition of nanoparticles in epoxy resin also influenced the dispersibility. Therefore, the dispersion and dosage of nano-Al_2_O_3_ and GO nanosheets in epoxy resin are the key factors to improve the performance of the composite coatings, so it is of great significance to solve the dispersion of nano-Al_2_O_3_ and GO nanosheets in epoxy resin. Furthermore, seven different hybrid ratios of RGO/Al_2_O_3_ were dispersed by ultrasonication into the epoxy matrix, and found that the synergy of RGO/Al_2_O_3_ at 6 : 4 enhanced the thermal, insulation and mechanical properties of epoxy resin. Zhou *et al.*^25^ studied ZrO_2_ nanoparticles were controllably anchored on the reduced-GO (rGO) nanosheets *via* an environmentally friendly single-step hydrothermal reaction and then incorporated into epoxy coatings to simultaneously improve the wear resistance and anti-corrosion performance of the coatings, The addition of 0.5 wt% ZrO_2_@rGO nanohybrids to the epoxy coating significantly improved the adhesion strength, impact resistance and hardness, and the average friction coefficient decreased by ∼42% and the wear resistance improved by ∼57.7%. The as-received ZrO_2_@rGO hybrid in epoxy coatings could effectively prevent electrolyte penetration, and ZrO_2_ particles played a synergistic role with rGO nanosheets in alleviating corrosion. ZrO_2_ particles uniformly grew on the surface of rGO nanosheets to form nanohybrids with good dispersity. Yu *et al.*^26^ reported that GO–Al_2_O_3_ hybrids were fabricated using GO as a precursor, then anchoring Al_2_O_3_ on GO sheets with 3-aminopropyltriethoxysilane. GO–Al_2_O_3_ hybrids not only achieved an homogeneous dispersion and compatibility in epoxy resin, but also exhibited an obvious superiority in reinforcing the anti-corrosion performance of epoxy coatings. However, the concentration of GO, Al_2_O_3_, and GO–Al_2_O_3_ hybrids were not optimized and deeply studied.

In this study, to reduce the agglomeration of nanoparticles in the resin, the silane coupling agent was used to modify nano-Al_2_O_3_ and GO, and the hybrid of nano-Al_2_O_3_ and GO nanosheets was also prepared. Then, the modified nanoparticles with different concentrations were dispersed into the water-borne epoxy resin by mechanical stirring. The dispersity and stability of nanoparticles in the epoxy resin were studied, and the hardness, abrasion and corrosion resistance of the composite coatings were further investigated and compared.

## 2 Experimental details

### 2.1 Raw materials

Nano-Al_2_O_3_ particles were purchased from Jiangsu Kexiang Anticorrosion Materials Co. The GO nanosheets (Industrial grade single-layer structure) were purchased from Suzhou Tanfeng Graphene Tech Co. Ltd, China. The following chemicals were purchased from Shanghai Nalle New Material Technology Co., the epoxy resin (A, the epoxy equivalent weight is 6002), curing agent (B, the active hydrogen equivalent weight is 591) and 37% hydrochloric acid (HCl). Silane coupling agents 3-aminopropyltriethoxysilane (KH-550) was provided by Chengdu Branch of Chinese academy of sciences. *N*,*N*-Dimethylformamide (DMF) was provided by Chengdu Long March Chemical Reagent Factory. The deionized water (D.I. water) was produced by a water purification machine (UPC-III-40L, Ulupure).

### 2.2 Surface modification

**Fig. 1:**
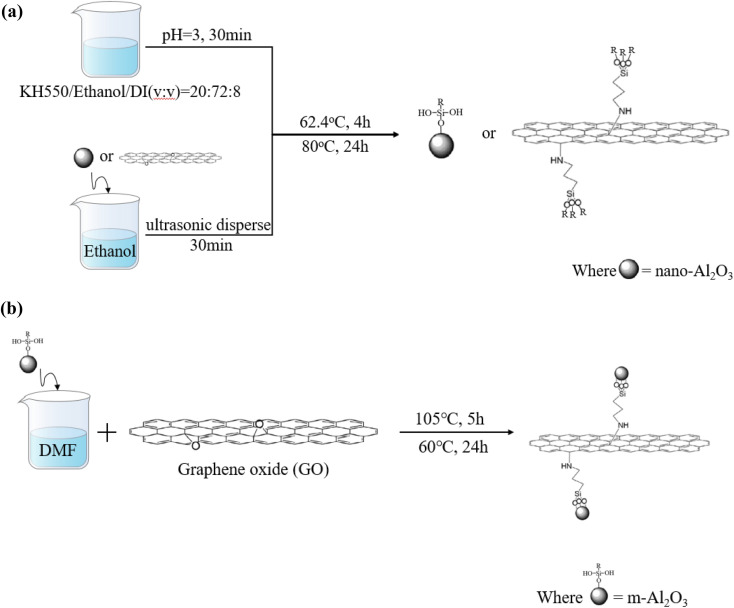
Illustration of the synthesis procedure for (a) m-Al_2_O_3_ or mGO and (b) m-Al_2_O_3_@GO hybrids.

The synthesis procedure for modified nanoparticles is shown in Fig. 1. For the m-Al_2_O_3_ or mGO (modified Al_2_O_3_ or modified GO), nano-Al_2_O_3_ or GO with 2 g weight was added into 50 mL anhydrous ethanol and then ultrasonically dispersed for 30 min. Meanwhile, the hydrolysis pretreatment osilane coupling agent was carried out. 20 mL KH-550, 72 mL anhydrous ethanol and 8 mL D.I. water were evenly mixed for 30 min and then the pH of the mixture solution was adjusted to 3.0 with 1.0 M HCl. Then, the mixture solution was treated while stirring using a mechanical stirrer at 60 °C for 4 h. After centrifugation process at a rotate speed of 1200 rpm for three times, every time was 10 min, and then washed with anhydrous alcohol and D.I. water, finally dried in a vacuum oven at 80 °C for 24 h to obtain functionalized m-Al_2_O_3_ or mGO nanoparticles, as shown in Fig. 1(a). As illustrated in Fig. 1(b), the m-Al_2_O_3_ nanoparticles with 0.1 g weight was dispersed in 250 mL *N*,*N*-dimethylformamide (DMF) to form a homogeneous suspension by mechanical stirring. GO with 0.4 g weight was added into the suspension *via* ultrasonication for 1 h. The mixture solution was reacted and stirred for 5 h at 85 °C, and the as-received m-Al_2_O_3_@GO hybrids were washed with anhydrous alcohol and D.I. water for three times in the centrifuge at a high rotate speed of 1200 r min^−1^, then dried at 60 °C in a vacuum oven for 24 h. These mixture resins filled with nanoparticles were divided into a glass bottle to observe the dispersity and stability of nanoparticles in expoy resin.

### 2.3 Preparation of the coating

The epoxy resin was dispersed with mGO, m-Al_2_O_3_ and mGO-Al_2_O_3_ hybrids by mechanical agitation at a rotate speed of 1300 r min^−1^ for 15 min to form a homogeneous dispersion system, and then taken into a constant temperature water bath at 60 °C until bubbles were removed in epoxy resin. Meanwhile, the size of the tinplate sheets was 70 × 17 × 3 mm^3^. Before adding the curing agent to the epoxy resin according to the volume ratio of 2 : 1, and then stirred slowly and uniformly for 10 min. The composite coatings were scraped on the tinplate sheets by a scraping rod. The thickness of the coating was controlled at around 70 ± 1 μm and dried naturally (see Fig. S1†). The as-prepared epoxy coatings were named m-Al_2_O_3_/epoxy, mGO/epoxy, mGO-Al_2_O_3_/epoxy, and neat epoxy coatings, respectively. The additional amount of nanoparticles is shown in Table 1. The modified nanoparticles were added according to the percentage of the total mass of epoxy resin and curing agent.

**Table 1:** The additional amount of nanoparticles

Name	Content				
Neat epoxy	0%				
m-Al_2_O_3_	1.5%	3.0%	4.5%	6.0%	7.5%
mGO	0.2%	0.4%	0.6%	0.8%	1.0%
m-Al_2_O_3_@GO	0.2%	0.4%	0.6%	0.8%	1.0%

### 2.4 Characterization

The microstructure and morphology of nanoparticles were obtained by scanning electron microscopy (SEM, JSM-6510, Japan). The crystal phases of the coating and particles were performed by X-ray power diffractometer (XRD, APEX II DUO) with Cu Kα radiation working in the Bragg–Brentano (θ–2θ) geometry utilizing a para-focusing geometry to increase intensity and angular resolution in the angle range of 6–78°. The chemical bonds and functional groups for modified nanoparticles were tested by the Fourier-transform infrared spectroscopy (FT-IR, Thermo Fisher spectrum, USA) spectra in the range from 400 to 4000 cm^−1^. The structure of the nanoparticles before and after modification was determined by a Thermodxr Raman Spectrometer with a 532 nm Raman laser. The surface morphology of the coatings was observed by a digital microscope (OM, VHX-700). Contact angles of the coatings were recorded using a goniometer (FM4000, KRUSS Germany). The liquid was distilled water, 5 μL in volume, which were dropped on the surface of the samples, in order to measure the contact angle. The final contact angle was determined three times at different places on the surface of the samples.

### 2.5 Hardness

The hardness of the coating was obtained by pencil hardness test, in accordance with the standard GB/T 6739-2006, and a pencil with a certain hardness was applied to the coatings. The scratches, indicated by the mark of the hard pencil, did not cause damage to the surface of the coatings.

### 2.6 Abrasion resistance

**Fig. 2:**
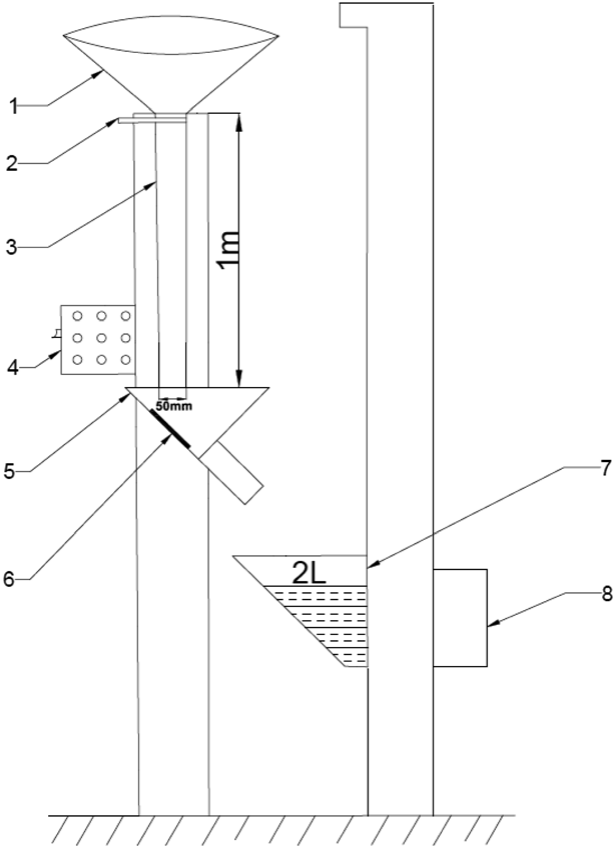
Schematic diagram of sand shaker (1-sand bucket; 2-sand drop switch; 3-sand drop tube; 4-total power; 5-sample pool; 6-sample; 7-sand bucket; 8-electric machinery).

The abrasion resistance of the coating was assessed by a sand shaker, and the schematic diagram of sand shaker is shown in Fig. 2. The fine sands with irregular prisms are 30# brown corundum with a bulk density of 1.87 g cm^−3^ and a particle density of 3.96 g cm^−3^. The sand washing height and a diameter of sand washing pipe were 1.0 m and 50 mm, respectively. The surface of the coating was subjected to sand punching strength, and the angle between the sample and the flow direction of sand was 45°. The fine sands were collected in sand bucket, and then reused. The flushing volume of sand for each time was 2 L.

### 2.7 Corrosion resistance

The opposite surface of the sample was glued with waterproof adhesive glue, and the exposed area was 10 × 30 mm^2^. Then, the samples were placed in the 3.5 wt% NaCl aqueous solution open to the air, simulating the seawater environment. According to the national Standard GB/T 1771-2007 ‘color paint and varnish-neutral salt fog performance test’, the composite coatings were placed in the salt spray test box (BGD881, Biuged, Guangzhou). The corrosion conditions were: 5 wt% NaCl aqueous solution, chamber temperature of 35 °C, saturation temperature of 47 °C, pH of 6.5–7.2 and spray volume of 1–2 mL/80 cm^2^ h^−1^. The angle between the tested surface of the sample and the vertical direction was fixed at 20°. The exposed area of the composite coatings was around 120 × 50 mm^2^.

The samples used as a working electrode was bonded with a conductive wire by a conductive adhesive and then covered with acrylic resin leaving a square surface area of 1 cm^2^ exposed to 3.5 wt% NaCl aqueous solution. The electrochemical workstation (CHE 660E) was used to test the open circuit potential (OCP) and polarization curves of the samples. A standard three-compartment cell was used with an Ag/AgCl 3 M KCl electrode and a Pt electrode as a reference and counter electrodes, respectively. The potentiodynamic current–potential curves were recorded at a sweep rate of 20 mV min^−1^. Before the polarization test, the electrochemical impedance spectroscopic (EIS) measurements were carried out at the measured steady-state OCP value of the corresponding working electrode in the frequency range of 10^−2^ to 10^5^ Hz. All the experiments were conducted at room temperature. All impedance measurements were made in Faraday cages to minimize external disturbances and the experimental data were fitted by ZsimDemo software. The corrosion rate (CR) of the composite coatings can be calculated by the following eqn (1):1
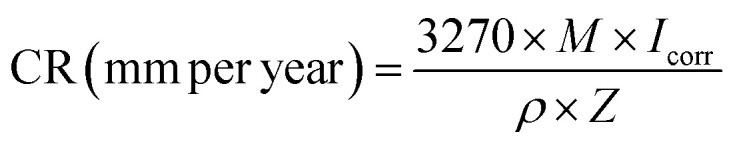
where *M* is the atomic mass of the metal (*M*_Fe_ = 55.845), *I*_corr_ is the corrosion current density (A cm^−2^), *ρ* is the density of the corroding material (*ρ* = 7.85 × 10^3^ kg m^−3^), and *Z* is the number of electrons transferred per metal atom (*Z* = 2).

## 3 Results and discussion

### 3.1 Characterization of nanoparticles

**Fig. 3:**
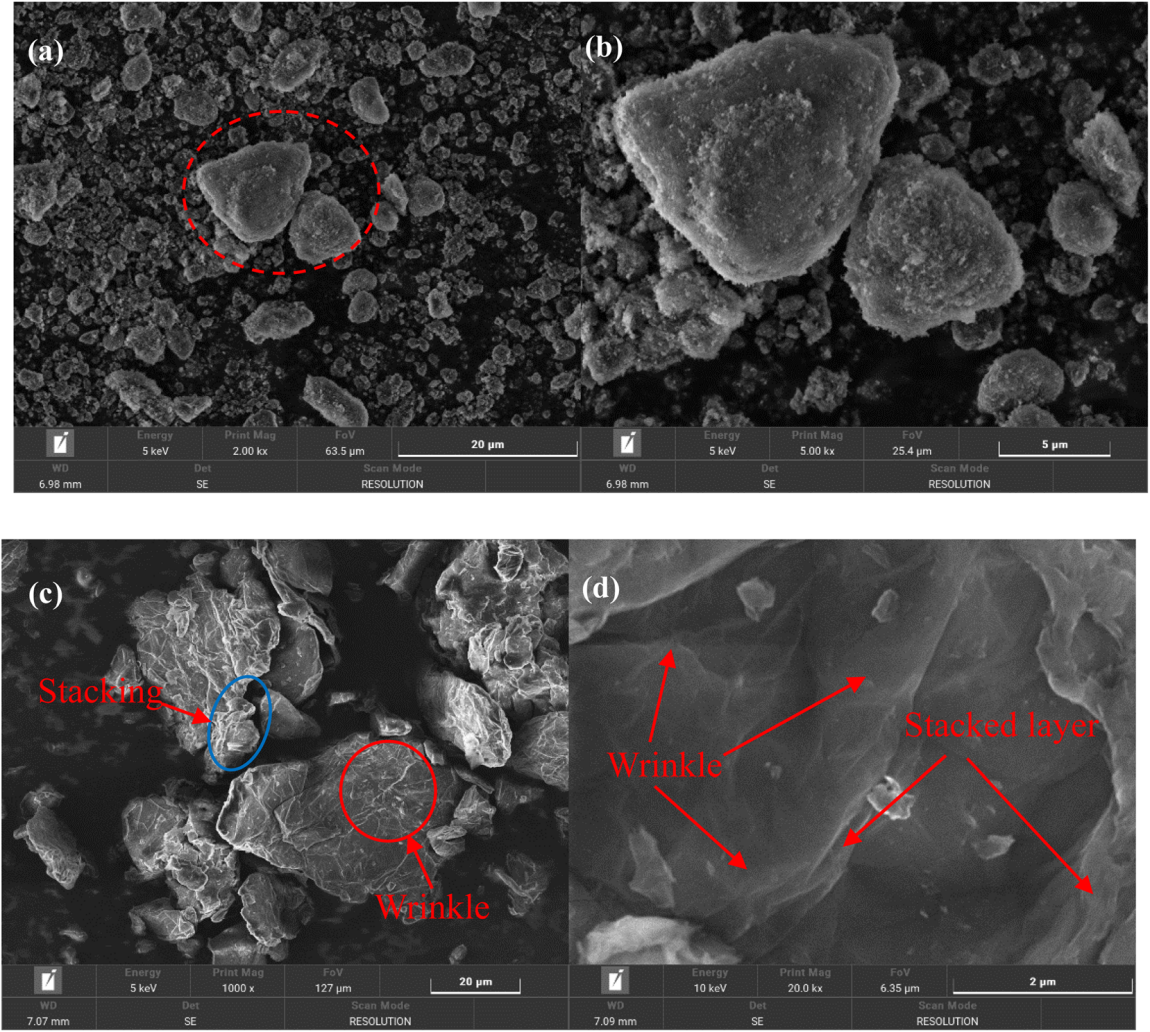
SEM images of nano-Al_2_O_3_ (a) and (b), GO nanosheets (c) and (d) low-magnification SEM images (a) and (c), and high magnification SEM images (b) and (d).

The morphology and microstructure of nano-Al_2_O_3_ and GO nanosheets are shown in Fig. 3. Nano-Al_2_O_3_ presents nearly spherical particles with significant agglomerations (Fig. 3(a) and (b)), which would influence the dispersion of particles in epoxy resin. There are some large layered particles (dotted red line), with high aspect ratio and good ability to restrain corrosive substances' penetration and diffusion. The wrinkles or wavy features are observed in a large area of GO plates, which present a stacked state (Fig. 3(c)). Accordingly, Fig. 3(d) depicts a high magnification SEM image of GO. The wrinkle morphology and several stacked layers of GO are observed. The GO leaves were firmly accumulated in the stacked form with a micrometer-scale width.

**Fig. 4:**
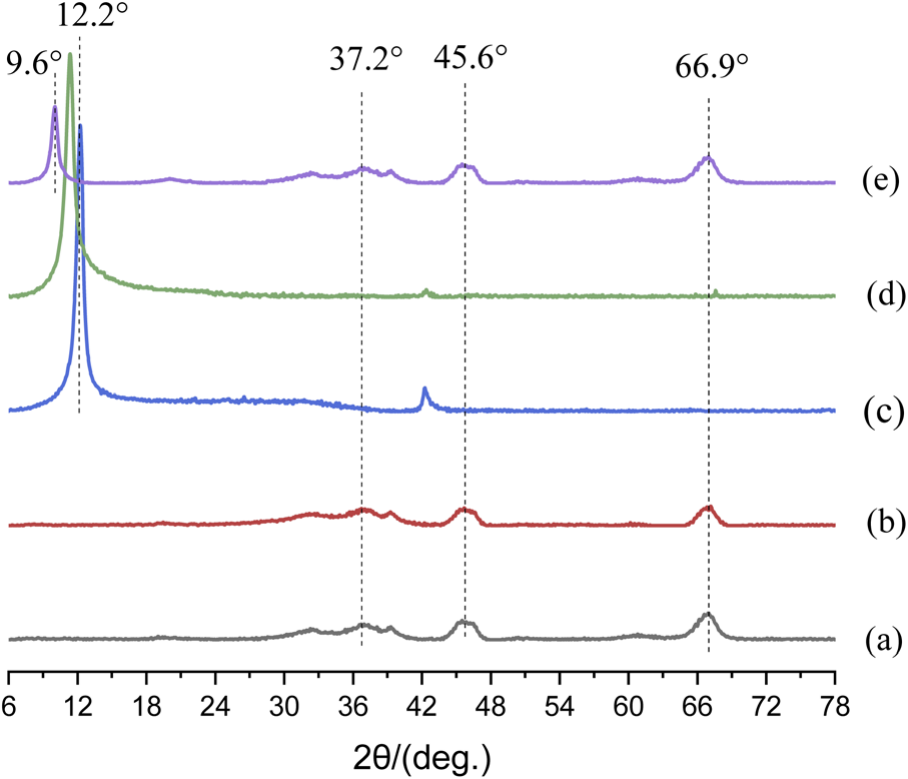
XRD patterns of (a) nano-Al_2_O_3_, (b) m-Al_2_O_3_, (c) GO and (d) mGO and (e) m-Al_2_O_3_@GO hybrids.

Fig. 4 shows the XRD patterns of nanoparticles. In Fig. 4(a) and (b), the diffraction peaks of 66.9°, 45.6°and 37.2° stemmed from the characteristic diffraction peak of γ-Al_2_O_3_,^27,28^ and the diffraction peak area of m-Al_2_O_3_ at 2-theta of 66.9° was significantly enhanced, which indicated that the content of crystalline phase increased. However, up to this point, we were still unable to determine whether the modification of nano-Al_2_O_3_ was successful. In Fig. 4(c) and (d), a broad diffraction peak at around 2-theta of 12.2° was corresponded to the reflection of GO.^28^ This is due to the oxygen-containing functional groups embedded in the interlayer spacing of GO nanosheets.^29^ There was a strong diffraction peak at 2-theta of 11.41° for mGO nanosheets, and a diffraction peak at 2-theta of 11.97° was observed for GO nanosheets. According to the Bragg eqn (2):^30^

2 2*d* sin *θ* = *nλ*

where *d* is the crystal plane spacing, *θ* is the Bragg angle, *λ* is the wavelength, and *n* is the reflection order. According to the eqn (2), the crystal plane spacing *d* of GO (100) and mGO (100) was 7.39 nm and 7.75 nm, respectively. The increase in interlayer space indicated that silane molecules and alkyl chains were successfully grafted onto the surface of GO nanosheets. Moreover, the diffraction peaks of nano-Al_2_O_3_ were also revealed in the XRD pattern of mGO@Al_2_O_3_ hybrids (Fig. 4(e)). In the case of GO, the diffraction peak at 12.2° slightly decreased to 9.6°, which indicated that GO nanosheets were sufficiently disordered and loosened by m-Al_2_O_3_ nanoparticles,^31^ but the nanosheet-like structure of GO was still retained during surface functionalization process. In the ESI of Fig. S2,† there are some strong diffraction peaks, corresponding to Sn layer on the steel (Fig. S2(a)–(c)),† because X-ray can strongly penetrate the composite coatings. There is a weak C(002) diffraction peak from the epoxy resin, however, there are no signals for the small amount of particles in the coatings.

**Fig. 5:**
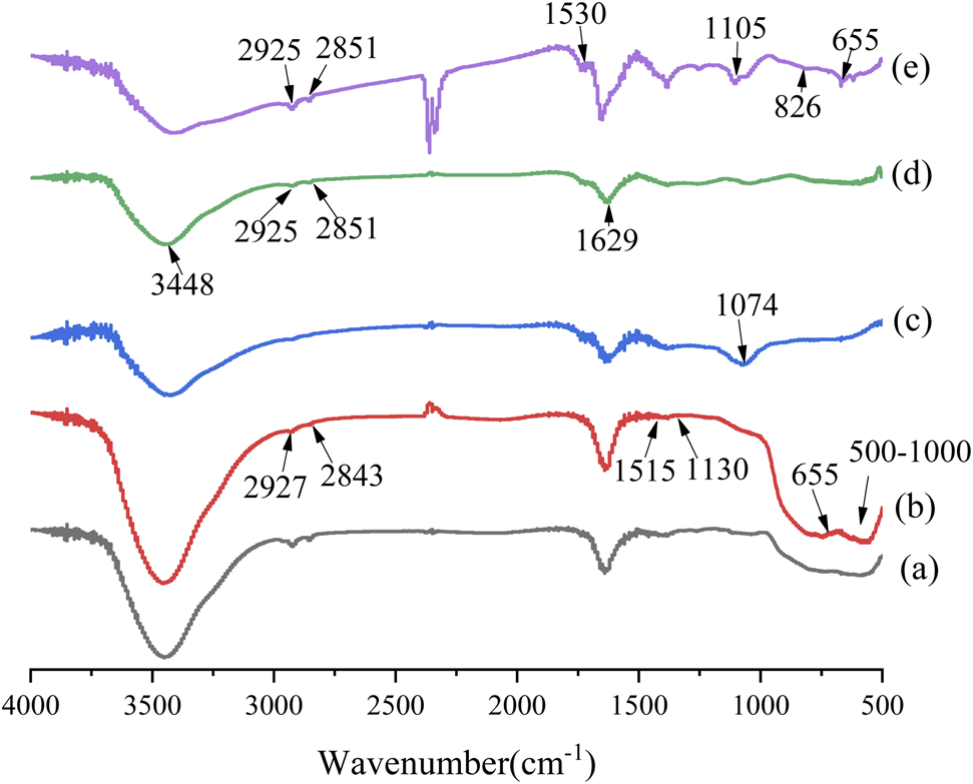
FT-IR spectra of (a) nano-Al_2_O_3_, (b) m-Al_2_O_3_, (c) GO, (d) mGO and (e) m-Al_2_O_3_@GO hybrids.

FT-IR spectra of the nanoparticles are shown in Fig. 5. Compared with nano-Al_2_O_3_ particles, the new characteristic peaks of m-Al_2_O_3_ nanoparticles after surface functionalization were observed at 2927 cm^−1^, 2843 cm^−1^ (–CH), 1515 cm^−1^ (N–H), and 1130 cm^−1^ (Si–O–Si),^32,33^ which were attributed to KH550, and importantly, the peak at 655 cm^−1^ belonged to Si–O–Al bonding,^34,35^ depicted in Fig. 5(a) and (b). It was indicated that the nano-Al_2_O_3_ particles were functionalized and modified by KH550 *via* chemical grafting. In Fig. 5(c) and(d), the characteristic absorption peaks of GO at 3448 cm^−1^ (–OH), 1629 cm^−1^ (C–OH) and 1074 cm^−1^ (C–O–C) were clearly observed,^36^ indicating that GO contained a large number of oxygen-containing hydrophilic groups and adsorbed water. The disappearance of the C–OH bonding vibration peak at 1074 cm^−1^ after KH550 surface modification was attributed to the reaction of KH550 with hydroxyl groups and the formation of Si–O–C bonding. Two new absorption peaks appeared at 2924 cm^−1^ and 2851 cm^−1^, which were the stretching vibration peaks on the methyl group and methylene group on the KH550.^37^ It was indicated that KH550 was grafted onto the chemical bonding of GO. For m-Al_2_O_3_@GO hybrids (Fig. 5(e)), the broad absorption band at ∼500–1000 cm^−1^ was assigned to the Al–O–Al group of nano-Al_2_O_3_ particles. Furthermore, the peak at 1530 cm^−1^ represented the secondary amide N–H-bending and C–N stretching, and N–H rocking at 826 cm^−1^ appeared while 1074 cm^−1^ (C–O–C) was absent, implying the reaction between the epoxide of GO and amino group of the KH550.^35,38,39^ The results showed a referential value for the effect of the interaction between m-Al_2_O_3_ nanoparticles and GO nanosheets to some extent.

**Fig. 6:**
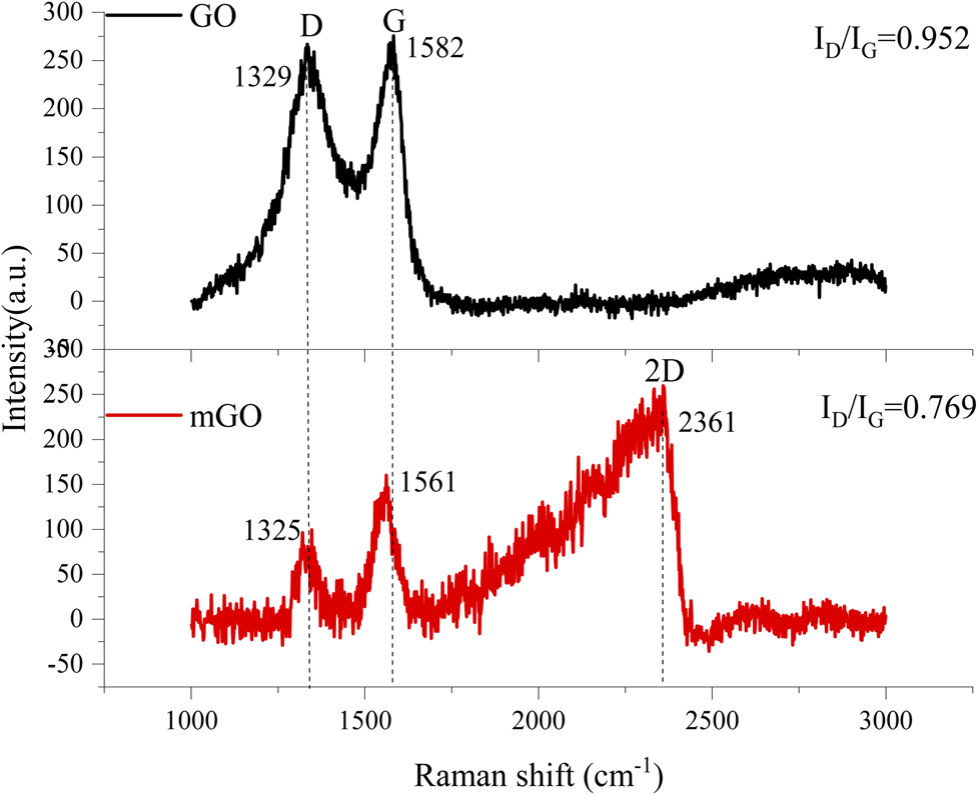
Raman spectra of GO and mGO.

Fig. 6 displays the Raman spectra of GO and mGO. G-band is originated from the first-order scattering of vibration modes of sp^2^-carbon atoms, D-band corresponds to the presence of vacancies or distortions in the carbon rings, and the 2D-band is valuable to predict the number of graphene layers.^40,41^ After surface modification, G- and D-bands were shifted to lower wavenumbers of 1561 cm^−1^ and 1325 cm^−1^, respectively. The appearance of D-band in Raman spectrum for GO nanosheets was due to the introduction of oxygen moieties between GO nanosheets in which most of the sp^2^ bonds were transformed into sp^3^ bonds. The destruction of structural tube symmetry of GO was due to the decrease in the size of the sp^2^ graphitic domains upon oxidation, leaving a disorganized and amorphous structure.^42,43^ The intensity ratio between D- and G-bands (*I*_D_/*I*_G_) is used to determine the structural integrity of graphene and its degree of disordering.^44^ After the surface functionalization of GO, the intensity ratio of mGO was 0.769, which was significantly lower than that of virgin GO (0.952). The vacancies and defects in the structure of GO were reduced after surface modification with KH550, and these vacancies and defects were caused by the reduction of oxygen functional groups.^24^ The intensity ratio of 2D-band and D-band is used to predict the number of GO layer. If the intensity ratio of 2D-band and D-band is high, the number of GO layer is few. Therefore, the number of mGO layer is much less than that of virgin GO, because of no evidence of 2D-band in Raman spectrum.^45^ This result was proved by SEM image in Fig. 3(c) and XRD result in Fig. 4(e).

### 3.2 Optical micrograph

**Fig. 7:**
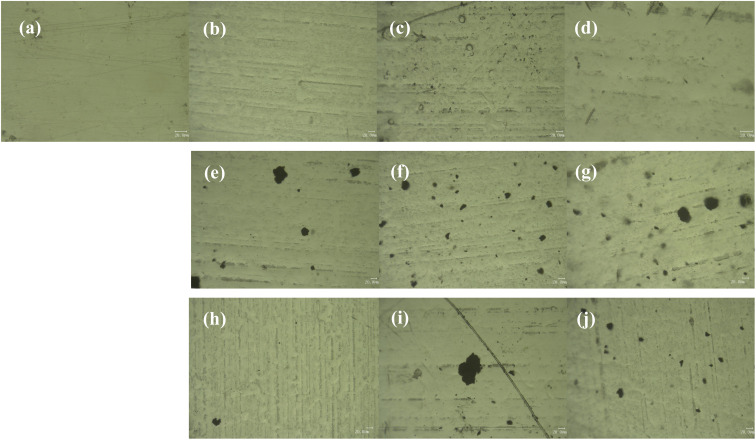
OM images of the coatings (a) EP, (b) 1.5 wt% m-Al_2_O_3_/epoxy, (c) 4.5 wt% m-Al_2_O_3_/epoxy, (d) 7.5 wt% m-Al_2_O_3_/epoxy, (e) 0.2 wt% mGO/epoxy, (f) 0.6 wt% mGO/epoxy, (g) 1.0 wt% mGO/epoxy, (h) 0.2 wt% m-Al_2_O_3_@GO/epoxy, (i) 0.4 wt% m-Al_2_O_3_@GO/epoxy, (j) 1.0 wt% m-Al_2_O_3_@GO/epoxy.

The OM images of the top-surface of the coatings are shown in Fig. 7. Fig. 7(a) shows the OM image of the neat epoxy coating, the surface is smooth without defects. With the increase of the amount of m-Al_2_O_3_, some particles gradually appear on the surface of the coating. As shown in Fig. 7(b), the strip depression of 1.5 wt% m-Al_2_O_3_/epoxy coating may be caused by poor compatibility with epoxy resin. A large number of m-Al_2_O_3_ particles are observed on the surface of 4.5 wt% m-Al_2_O_3_/epoxy coating (Fig. 7(c)). On the surface of 7.5 wt% m-Al_2_O_3_/epoxy coating (Fig. 7(d)), a small amount of unobvious particle aggregation can be observed. The surface of the coatings filled with high content of Al_2_O_3_ was not smooth (see Fig. S3(a) and (b)),† compared with the coating with low content of particles. It can be seen that with the increase of the amount of mGO nanosheets, from Fig. 7(e)–(g), the number of nanosheets in the coating is obviously increased, but the particle sizes are different, and there are large pieces of mGO that are not completely dispersed, as shown in Fig. S3(c) and (d),† affecting the performance of the coating. When 0.2 and 0.4 wt% m-Al_2_O_3_@GO particles were added, the hydrides on the surface of the coating are evenly distributed and occasionally large particles existed (Fig. 7(h) and S3(e)†) It is observed from Fig. 7(i) that there are irregular black flakes with large diameter, which is the accumulation of GO nanosheets without dispersion. When the addition amount of m-Al_2_O_3_@GO hybrid was 0.8 and 1.0 wt% (Fig. S3(f) and 7(j)), the particle size is different, and it is evenly distributed in the composite coating.

### 3.3 Contact angle

**Fig. 8:**
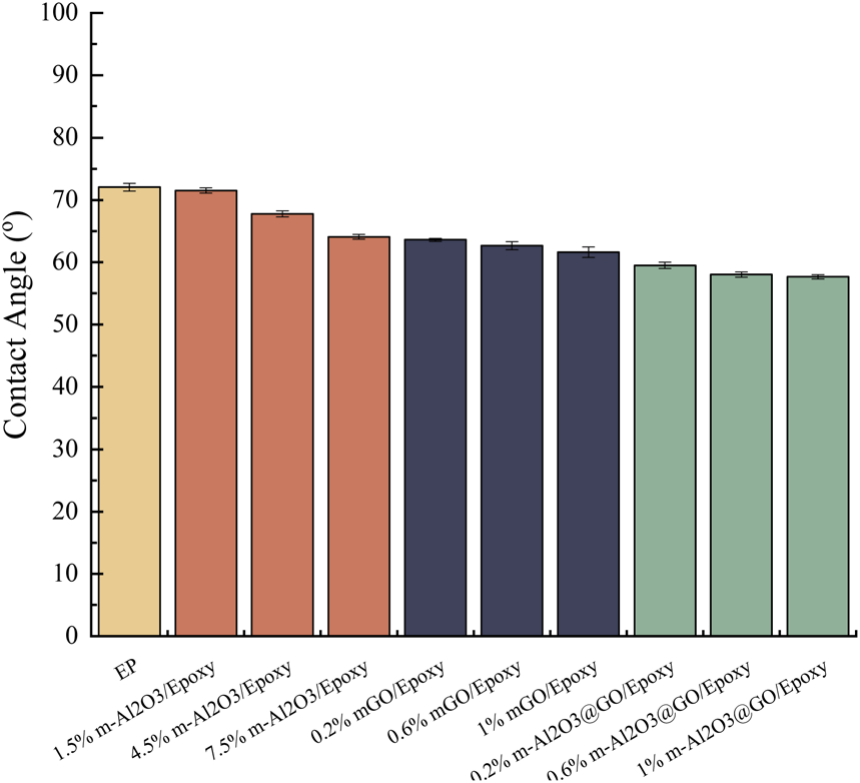
Contact angles of the coatings.

The contact angles of the composite coatings are shown in Fig. 8 and S4.† The wettability of the neat epoxy coating is poor, because the contact angle is the largest, about 72.05°, which is hydrophilic. With the increase of the content of m-Al_2_O_3_ nanoparticles, the contact angle decreases and the hydrophilicity improves. The addition of mGO and hybrid will also further reduce the contact angle of the coating, and the effect of these two additions is much more obvious than that of m-Al_2_O_3_, 1.0 wt% m-Al_2_O_3_@GO/epoxy has the smallest contact angle and the largest hydrophilicity, with a contact angle of 57.65°. Al_2_O_3_ itself is hydrophobic,^59^ which verifies that Al_2_O_3_ particles have been successfully modified by KH550 from hydrophobic to hydrophilic. FT-IR shows that GO contained a large number of oxygen-containing hydrophilic groups and adsorbed water, as a result, the hydrophilicity of GO and Al_2_O_3_ is improved after hybridization.

### 3.4 Dispersibility and stability

**Fig. 9:**
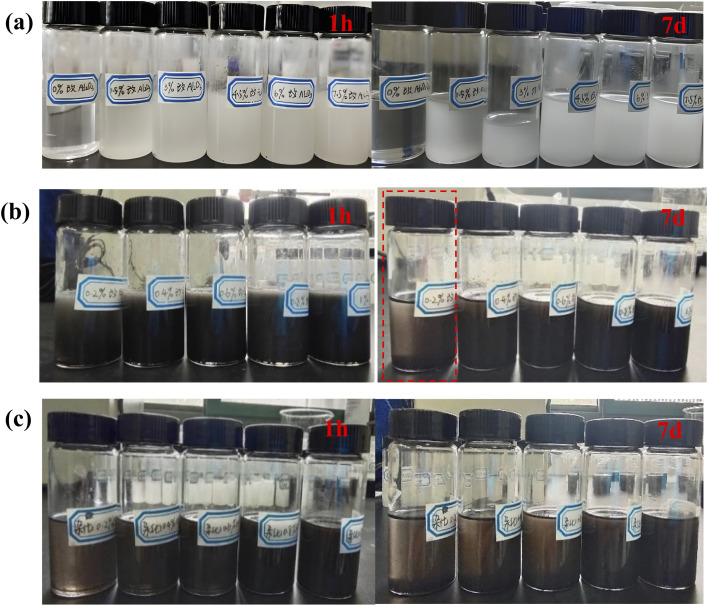
Dispersibility and stability of nanoparticles with different contents in epoxy resin for different holding times, (a) m-Al_2_O_3_, (b) mGO, and (c) m-Al_2_O_3_@GO hybrids.

Fig. 9 shows the dispersibility and stability of nanoparticles with different contents in water-borne epoxy resin. By observing and comparing the dispersibility and stability of nanoparticles immersed in the epoxy resin after several days, it can be seen from Fig. 9(a) that all the epoxy resin filled with m-Al_2_O_3_ was still an uniform suspension. The m-Al_2_O_3_ nanoparticles were well dispersed in epoxy resin and kept stable for a long time. As the holding time increased (Fig. 9(b)), the delamination of the mGO/epoxy became more pronounced. On the 7th day, 0.2 wt% mGO in the epoxy showed the evidence of delamination (see the red dotted line). After holding for 55 days, almost all of the mGO fillers had settled at the bottom of the glass bottles (See Fig. S5).† In comparison, 1.0 wt% mGO in the epoxy had the best stability. In Fig. 9(c), the delamination of m-Al_2_O_3_@GO in the epoxy was even worse than that of mGO in the resin, because the GO nanosheets in the hybrids were not modified, resulting in the poor dispersibility and stability. After holding for 24 h, 0.2 wt% m-Al_2_O_3_@GO/epoxy gradually delaminated (See Fig. S5),† and on the 7th day the hybrid fillers delaminated more obviously while 0.4 wt% and 0.6 wt% m-Al_2_O_3_@GO/epoxy also began to delaminate. On the 55th day, almost all hybrid nanofillers were precipitated at the bottom of the bottle and the dispersion of 1.0 wt% m-Al_2_O_3_@GO/epoxy was better (Fig. S5),† in comparison. The dispersibility and stability of nanoparticles in the epoxy resin have a significant influence on the performance and properties of the composite coatings.^46^

### 3.5 Hardness

**Fig. 10:**
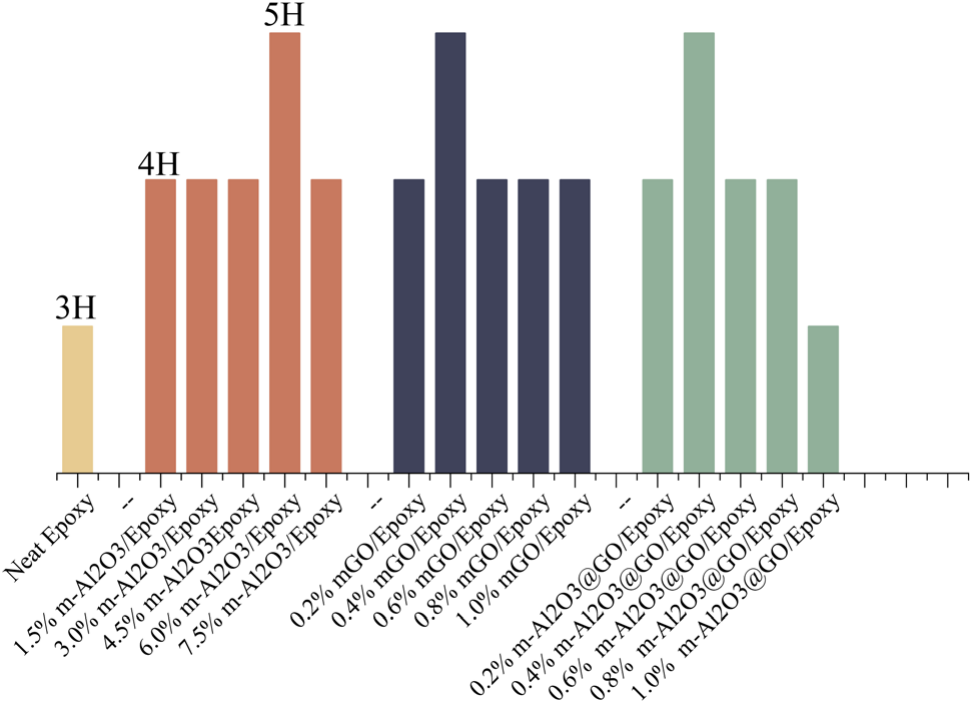
Hardness of the coatings.

Fig. 10 shows the variations in the hardness of the composite coatings. The hardness of the neat epoxy was 3H. With the addition of modified nanoparticles, the hardness of the composite coatings increased from 3H to 4H while the hardness of the coatings doped with 6.0 wt% m-Al_2_O_3_, 0.4 wt% mGO and 0.4 wt% m-Al_2_O_3_@GO hybrids increased by two grades. The main reason was that when the nanoparticles were added to the epoxy resin, m-Al_2_O_3_ nanoparticles formed a tight grid structure with the epoxy, so that the composite coatings have high hardness. In addition, the modified nanoparticles were surrounded by a large number of epoxy functional groups, which can bond with the amine curing agent added to the coating and enhance the bonding force between the molecules. However, as the addition amount of m-Al_2_O_3_@GO hybrid was increased to 1.0 wt%, the hardness of the coating was reduced to 3H due to the agglomeration of nanoparticles, resulting in the weakness in the adhesion between the epoxy matrix and nanoparticles.^47,48^ The adhesion of the coatings on the substrates was assessed by cross-cut testing with 3M tape, as shown in Table S1.† In contrast, the addition amount of mGO and m-Al_2_O_3_@GO hybrids was less than that of m-Al_2_O_3_ for the same hardness values of the coating.

### 3.6 Abrasion resistance

**Fig. 11:**
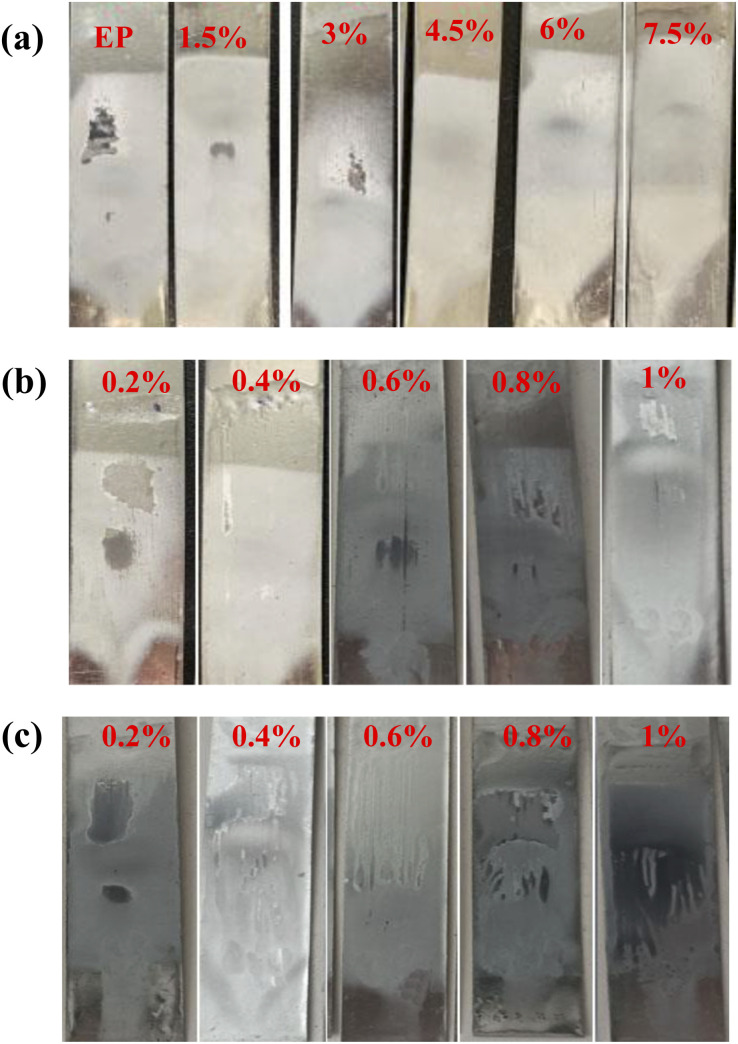
Digital images of impact resistance of composite coatings after sand punching (a) m-Al_2_O_3_/epoxy, (b) mGO/epoxy, and (c) m-Al_2_O_3_@GO/epoxy composite coatings.

Fig. 11 displays the digital images of the composite coatings after sand punching. The abrasion resistance of the coating is assessed and evaluated in Table 2. As shown in Fig. 11 and Table 2, when m-Al_2_O_3_ particles were added to the epoxy, the abrasion resistance of the composite coatings was better than that of the neat epoxy coating, with sand flushing for several times and small fracture area can be observed. When the addition amount of m-Al_2_O_3_ particle was 1.5 wt%, the abrasion resistance of the coating was greatly improved (see Fig. 11(a)) because m-Al_2_O_3_ particle with 1.5 wt% content had a good dispersibility in epoxy resin. When the modified nanoparticles were filled in epoxy resin, several molecular chains passed through the surface of a nanoparticle to form physical cross-linking points. The nanoparticles with good dispersion in the epoxy resin effectively filled the micropores or pinholes in the coating, thereby reducing the defect density in the coatings. The nanoparticles adsorbing macromolecular chains can play the role of evenly distributing the load. When the surface of the coating was subjected to external force, it can provide a large surface area for it through cross-linked nanoparticles, to adsorb more molecular chains, to greatly homogenize the external stress distribution, and to reduce the friction stress in local areas and finally to effectively improve the wear resistance of the coating. In general, the addition of m-Al_2_O_3_ nanofillers in the epoxy resin can not only improve the abrasion resistance of the coating but also reduce the fracture area on the surface of the coating. However, the abrasion resistance of the coating is not improved with increasing amount of m-Al_2_O_3_ compared to the epoxy reinforced with 1.5 wt% m-Al_2_O_3_, which is attributed to the agglomeration of nanoparticle in the epoxy doped with high concentration of the nanoparticles. Loading of nanoparticle is another parameter that might explain the deteriorating effect of abrasion resistance of the coatings because it increases the probability of agglomeration of particles in the composite coating, increasing the intensity of stress concentration in the composite coatings.^47^

**Table 2:** Abrasion resistance of composite coatings with nanofillers

Coating	Sand volume/L	Sand punching times	Evaluation
Neat epoxy (EP)	4(broken)	2	Medium
1.5 wt% m-Al_2_O_3_	12(broken)	6	Excellent
3.0 wt% m-Al_2_O_3_	4(broken)	2	Medium
4.5 wt% m-Al_2_O_3_	8(unbroken)	4	Good
6.0 wt% m-Al_2_O_3_	4(unbroken)	2	Medium
7.5 wt% m-Al_2_O_3_	4(unbroken)	2	Medium
0.2 wt.%mGO	4(broken)	2	Good
0.4 wt.%mGO	2(broken)	1	Medium
0.6 wt.%mGO	2(unbroken)	1	Good
0.8 wt.%mGO	2(unbroken)	1	Good
1.0 wt.%mGO	2(broken)	1	Poor
0.2 wt% m-Al_2_O_3_@GO	2(broken)	1	Poor
0.4 wt% m-Al_2_O_3_@GO	2(less cracking)	1	Medium
0.6 wt% m-Al_2_O_3_@GO	2(broken)	1	Poor
0.8 wt% m-Al_2_O_3_@GO	2(broken)	1	Poor
1.0 wt% m-Al_2_O_3_@GO	4(broken)	2	Good

The agglomeration of mGO and m-Al_2_O_3_@GO nanoparticles occurred in the coating, which made poor abrasion resistance of the coatings, compared with m-Al_2_O_3_/epoxy coating (Fig. 11(b) and (c)). In Fig. 11(b), the surface of 0.1 wt% mGO/epoxy coating presents some damaged areas after sand punching for four times. However, the surface of the coating with the addition of excess mGO exhibited poor abrasion resistance, due to the agglomeration of the high-content mGO nanosheets in the epoxy resin by mechanical stirring. GO has excellent lubrication performance because of its unique two-dimensional structure, however, the dispersion of mGO in the epoxy influences the abrasion resistance of the composite coatings. According to the results of above sub-section, the dispersibility and stability of mGO and m-Al_2_O_3_@GO in the epoxy are much poor than that of m-Al_2_O_3_ particles in the epoxy. In Fig. 11 and Table 2, the abrasion resistance of mGO/epoxy and m-Al_2_O_3_@GO/epoxy composite coatings were much lower than that of m-Al_2_O_3_/epoxy coatings, with large damaged area on the surface of the coating. 0.2 wt% mGO/epoxy and 1.0 wt% m-Al_2_O_3_@GO/epoxy coatings had relatively good abrasion resistance. For m-Al_2_O_3_@GO/epoxy coating, the abrasion resistance is not the best, the hybrids can not play the role of the rolling bearing and friction transfer by mechanical stirring process.^23^

### 3.7 Corrosion resistance

#### 3.7.1 Seawater corrosion

The coatings were immsered in 3.5 wt% NaCl solution for a long-term period (see Fig. S5).† After the immersion of 62 days, the surface of the coatings showed different corrosion phenomena. In Fig. S6(a),† the surface of the coatings doped with and without m-Al_2_O_3_ was good, with no evidence of delamination and pitting corrosion. However, the corrosion products were observed along the side of samples (see the red circled area), due to no protection by the red glue for the coating. In Fig. S6(b),† the surface of the coatings filled with 0.6 wt% and 0.8 wt% mGO was composed of yellow corrosion production, and the delamination of the coating was observed (see the red line), which was attributed to the defects, such as micropores and pinholes in the coating. Some micropores were present on the surface of the coatings filled with 0.6 wt% mGO, resulting in the pitting corrosion. On the other hand, this may be attributed to the agglomeration of mGO in the epoxy, resulting in the direct corrosion along the conductive mGO nanosheets with agglomeration. The less 0.6 wt% mGO/epoxy coatings showed good corrosion resistance. The same results were taken from the 0.6 wt% and 0.8 wt% m-Al_2_O_3_@GO/epoxy coatings. In Fig. S6(c),† 0.6 wt% m-Al_2_O_3_@GO/epoxy coating had poor corrosion resistance, but the corrosion resistance of the coatings with less 0.6 wt% m-Al_2_O_3_@GO was relatively good. The pitting corrosion (see the red circled area) was observed on the surface of the coatings. The formation of the corrosion rust involved several redox reactions (3)–(6):^49^

3 Fe → Fe^2+^ + 2e^−^

4 Fe^2+^ → Fe^3+^ + 2e^−^

5 O_2_ + 2H_2_O + 4e^−^ → 4OH^−^

6
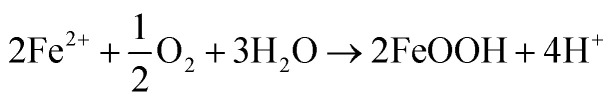


According to the above discussion, the corrosion resistance of the coating with high-content m-Al_2_O_3_ particle was better than that of the coating with low-content mGO nanosheets and m-Al_2_O_3_@GO hybrids. For the composite coatings filled with mGO nanosheets and m-Al_2_O_3_@GO hybrids, the coatings with less content of fillers showed relatively good corrosion resistance, due to the good dispersibility and stability of the low-content nanofillers in the epoxy resin.

#### 3.7.2 Salt spray corrosion

**Fig. 12:**
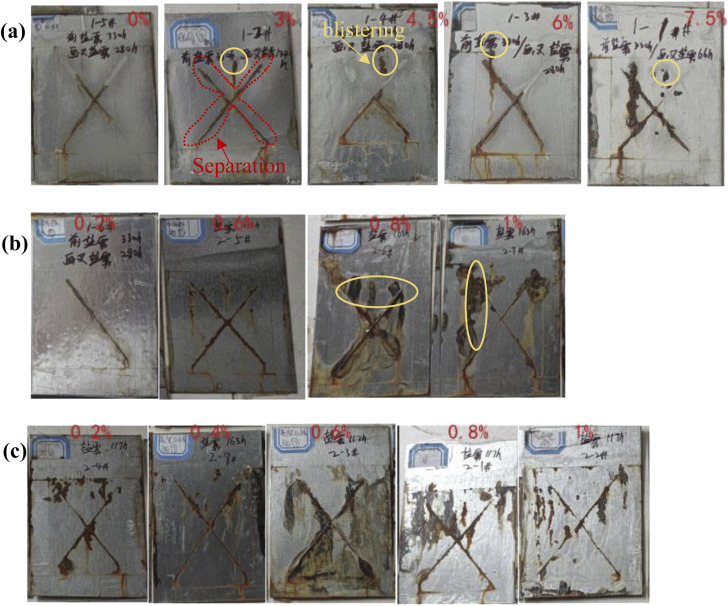
Digital images of the composite coatings containing (a) m-Al_2_O_3_, (b) mGO and (c) m-Al_2_O_3_@GO hybrids after exposure to salt spray test.

Fig. 12 shows the digital images of the coatings exposed to salt spray corrosion environment. Table 3 summarizes the results and evaluation. At the first stage, these samples were kept stale in salt spray conditions for 330 h before the samples were not scratched on the surface. At the next stage, after the surface of the coatings was scratched, the blistering and corrosion products were observed around on the scratch and other areas after 280 h, indicating the substrates were severely corroded. 4.5 wt% and 6.0 wt% m-Al_2_O_3_/epoxy composite coatings had better corrosion resistance, in comparison to the other coatings (Fig. 12(a) and Table 3). Some blisters with >2.0 mm rust width are observed for 7.5 wt% m-Al_2_O_3_/epoxy coating after 280 h. In Fig. 12(b), for the coatings filled with 0.2 wt% and 0.6 wt% mGO, the formation of corrosion products was limited to the scratch area and no delamination occurred while the significant damage, delamination and corrosion products were generated along the scratch area and beneath the coatings doped with 0.8 wt% and 1.0 wt% mGO, which was attributed to the poor dispersion of mGO nanosheets and conductive pathways for the agglomeration of mGO. Based on the salt spray duration, it can be inferred that 0.2 wt% mGO/epoxy coating had the best corrosion resistance. Meanwhile, GO itself is conductive, accelerating the electrolyte propagated in the coating through the scratches.^50^ In Fig. 12(c), for the m-Al_2_O_3_@GO/epoxy coatings, 0.4 wt% m-Al_2_O_3_@GO/epoxy coating corroded after 163 h had better corrosion resistance, in comparison. The surface of m-Al_2_O_3_@GO/epoxy coating was composed of blistering, pitting corrosion and a large number of corrosion products along with scratches. The corrosion products were black substances and yellow iron rust. The black substances were taken from the oxidation of the tin layer on the surface of the steel, due to the formation of black stannous oxides. Compared with the coatings doped with nanofillers, 4.5–6.0 wt% m-Al_2_O_3_/epoxy and 0.2–0.6 wt% mGO/epoxy composite coatings exhibited better corrosion resistance. However, the composite coatings with nanofiller hybrids did not have good corrosion resistance, even much poorer than neat epoxy coating. It can be inferred that m-Al_2_O_3_@GO nanohybrids by mechanical stirring were not beneficial to improve the corrosion resistance of the epoxy coating. The deep discussion will be addressed subsequently.

**Table 3:** Corrosion resistance for the coatings doped with different nanofillers after salt spray test^a^

Coating	Salt spray time	Salt spray effect	Evaluation
Neat epoxy	330 h* + 280 h**	Coating peeled off, no blistering, the rust width was more than 2 mm	Good
3.0% m-Al_2_O_3_	330 h + 134 h	One or two blistering with more than 2 mm rust width	Medium
4.5% m-Al_2_O_3_	330 h + 280 h	Coating peeled off, one or two blistering with more than 2 mm rust width	Good
6.0% m-Al_2_O_3_	330 h + 280 h	Coating peeled off, no blistering, the rust width was more than 2 mm	Good
7.5% m-Al_2_O_3_	330 h + 66 h	Many blistering with more than 2 mm rust width	Poor
0.2% mGO	330 h + 280 h	A few blistering	Good
0.6% mGO	0 h + 117 h	One or two blistering with more than 2 mm rust width	Medium
0.8% mGO	0 h + 163 h	Coating peeled off, severe corrosion	Poor
1.0% mGO	0 h + 163 h	Coating peeled off, severe corrosion	Poor
0.2% m-Al_2_O_3_@GO	0 h + 117 h	Many blistering with more than 2 mm rust width	Poor
0.4% m-Al_2_O_3_@GO	0 h + 163 h	One or two blistering with 2 mm rust width	Medium
0.6% m-Al_2_O_3_@GO	0 h + 117 h	Coating peeled off, severe corrosion	Poor
0.8% m-Al_2_O_3_@GO	0 h + 117 h	Many blistering with more than 2 mm rust width, severe corrosion	Poor
1.0% m-Al_2_O_3_@GO	0 h + 117 h	Many blistering with more than 2 mm rust width	Poor

a Note: *:salt spray test for the unscratched sample, and ** salt spry test for the scratched sample.

#### 3.7.3 Electrochemical corrosion

**Fig. 13:**
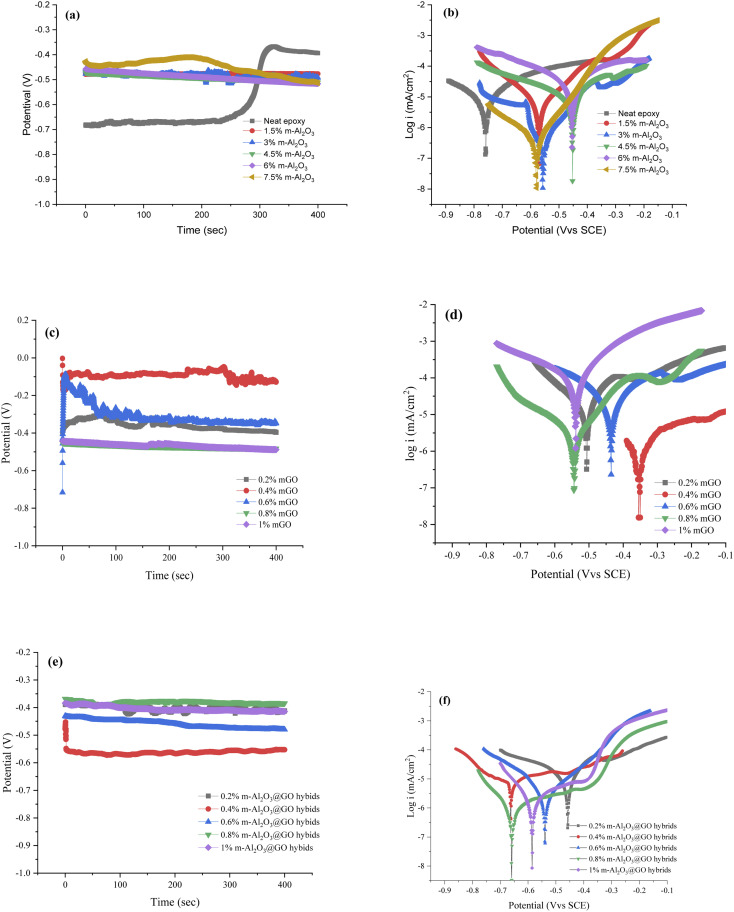
Open circuit voltage and potentiodynamic polarization curves of the coatings. (a) and (b) m-Al_2_O_3_/epoxy coating, (c) and (d) mGO/epoxy coating, (e) and (f) m-Al_2_O_3_@GO/epoxy coating.

Fig. 13 shows the open circuit voltage (OCP) and potentiodynamic polarization curves of the coatings. The electrochemical corrosion parameters of the coatings are summarized in Table 4. As the OCP value changed continuously with the increase of testing time. The OCP curves of the coatings for a long immersion time in 3.5 wt% NaCl solution are shown in Fig. 13(a). The OCP value of the neat epoxy coating was the smallest, about −0.59 V. However, the OCP value of the neat epoxy coating was unstable, which increased from −0.59 V to −0.38 V with increasing soaking time. The OCP values of the composite coatings were relatively stable, −0.45 to −0.5 V. It was indicated that the m-Al_2_O_3_/epoxy coating significantly increased the potential for more positive value. As a consequence, the composite coatings displayed better corrosion resistance than the neat epoxy coating. Fig. 13(b) depicts the polarization curves of the m-Al_2_O_3_/epoxy coatings. For the neat epoxy coating, the corrosion potential and corrosion current density were −0.76 V and 1.24 × 10^−5^ A cm^−2^, respectively. When 1.5 wt% m-Al_2_O_3_ fillers were added, the corrosion potential of the coating increased up to −0.61 V and its corrosion current density decreased to 6.97 × 10^−6^ A cm^−2^. The electrochemical impedance value of 1.5 wt% m-Al_2_O_3_/epoxy coating was the largest (Table 4). Generally, the coatings with high corrosion potential, low corrosion current density and large impedance exhibited good anti-corrosion properties.^51^ Therefore, in this work, the corrosion resistance of the composite coating was enhanced by the increase in the additional amount of m-Al_2_O_3_. However, excessive addition of nanofillers led to poor dispersion and the decrease in the corrosion resistance of the coatings. Among these coatings, 1.5 wt% m-Al_2_O_3_/epoxy coating exhibited good corrosion resistance.

**Table 4:** Electrochemical corrosion parameters of the coatings

Coating	*E*_OCP_/V	*E*_corr_/V	*I*_corr_/A cm^−2^	*R*_p_/Ω cm^2^	CR/mm per year
Neat epoxy	−0.590	−0.760	1.235 × 10^−5^	3375.4	1.436 × 10^−4^
1.5% m-Al_2_O_3_	−0.476	−0.610	6.972 × 10^−6^	428512.5	8.108 × 10^−5^
3.0% m-Al_2_O_3_	−0.483	−0.557	8.388 × 10^−7^	39728.4	9.755 × 10^−6^
4.5% m-Al_2_O_3_	−0.493	−0.452	1.502 × 10^−5^	3211.3	1.747 × 10^−4^
6.0% m-Al_2_O_3_	−0.490	−0.454	3.435 × 10^−5^	1265.2	3.995 × 10^−4^
7.5% m-Al_2_O_3_	−0.451	−0.579	5.540 × 10^−7^	47331.1	6.443 × 10^−6^
0.2% mGO	−0.359	−0.507	3.554 × 10^−5^	1211.0	4.133 × 10^−4^
0.4% mGO	−0.127	−0.354	1.697 × 10^−6^	22383.5	1.974 × 10^−5^
0.6% mGO	−0.348	−0.435	3.237 × 10^−5^	1216.2	3.765 × 10^−4^
0.8% mGO	−0.471	−0.544	3.044 × 10^−6^	7821.4	3.540 × 10^−5^
1.0% mGO	−0.467	−0.539	1.242 × 10^−4^	282.0	1.444 × 10^−3^
0.2% m-Al_2_O_3_@GO	−0.402	−0.458	3.466 × 10^−5^	1548.1	4.031 × 10^−4^
0.4% m-Al_2_O_3_@GO	−0.586	−0.539	3.752 × 10^−6^	5215.8	4.364 × 10^−5^
0.6% m-Al_2_O_3_@GO	−0.562	−0.659	1.384 × 10^−6^	22782.1	1.610 × 10^−5^
0.8% m-Al_2_O_3_@GO	−0.380	−0.662	2.786 × 10^−5^	2035.6	3.240 × 10^−4^
1.0% m-Al_2_O_3_@GO	−0.460	−0.586	1.780 × 10^−6^	16569.7	2.070 × 10^−5^

In Fig. 13(c), the OCP value of 0.4 wt% mGO/epoxy coating was the most positive, about −0.1 V. However, the OCP value of the coatings with high-content mGO nanosheets exhibited the large negative value, about −0.5 V, indicating the corrosion resistance of the coatings with a high concentration of mGO nanosheets was poor. In Table 4, 0.4 wt% mGO/epoxy coating had the positive corrosion potential, the smallest current density and the largest impedance, compared with the other coatings. Fig. 13(d) also shows the same result. Therefore, 0.4 wt% mGO/epoxy coating exhibited good corrosion resistance.

In Fig. 13(e), 0.4 wt% m-Al_2_O_3_@GO/epoxy coating had a negative OCP value, about −0.586 V. However, the OCP values of 0.2 wt% and 0.8 wt% m-Al_2_O_3_@GO/epoxy coatings were almost the same, about −0.4 V. In Fig. 13(f) and Table 4, 0.6 wt% m-Al_2_O_3_@GO/epoxy coating had the lowest current density and the largest impedance, although the corrosion potential was not positive. 0.2 wt% m-Al_2_O_3_@GO/epoxy coating had a positive OCP value, and the lowest impedance value. Therefore, 0.6 wt% m-Al_2_O_3_@GO/epoxy coating exhibited good corrosion resistance in m-Al_2_O_3_@GO/epoxy composite coatings. For the three kinds of composite coatings, the corrosion rates of the neat epoxy coating and the composite coatings filled with 1.5 wt% m-Al_2_O_3_, 0.4 wt% mGO and 0.6 wt% m-Al_2_O_3_@GO were 1.436 × 10^−4^ mm per year, 8.985 × 10^−7^ mm per year, 1.974 × 10^−5^ mm per year, and 1.610 × 10^−5^ mm per year, respectively. Therefore, the corrosion resistance of the coatings is sorted in the order of 1.5 wt% m-Al_2_O_3_/epoxy coating > 0.6 wt% m-Al_2_O_3_@GO/epoxy coating > 0.4 wt% mGO/epoxy coating > the neat epoxy coating.

**Fig. 14:**
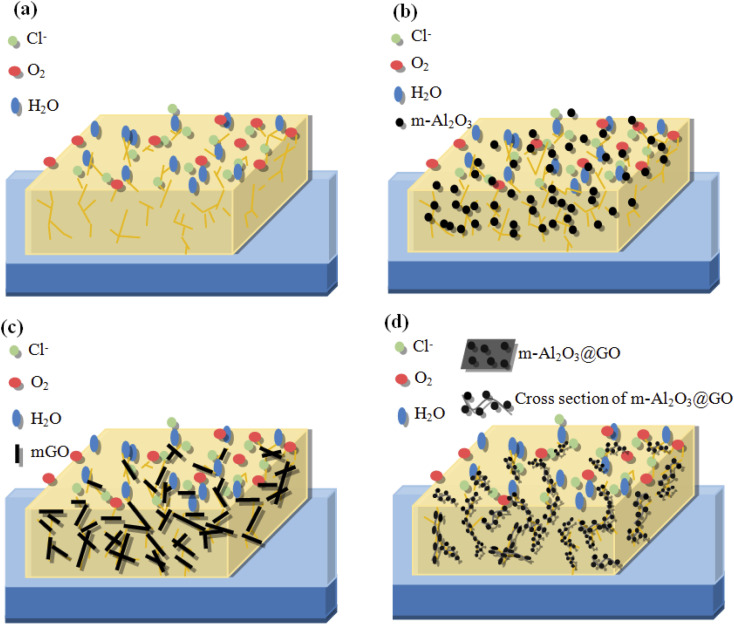
Schematic diagrams of (a) neat epoxy coating, (b) m-Al_2_O_3_/epoxy coating, (c) mGO/epoxy coating and (d) m-Al_2_O_3_@GO/epoxy coating.

Monetta *et al.*^52^ suggested that the corrosion failure of the epoxy coatings occurred in two steps. The first step was related to water uptake into the epoxy coating, while the second step was related to the diffusion of Cl^−^ ions through the coating. The epoxy network with a high crosslinking density of polymer was affected by corrosion medium, micropores or pinholes in the epoxy caused corrosion medium H_2_O, O_2_ and Cl^−^ ions to penetrate the interface between the metal and the epoxy coating, then the surface of the tinplate substrate was corroded (Fig. 14(a)). In addition, the molecular chain of the epoxy coating was hydrolyzed and degraded, which was easy to form defects, such as microcracks, and the corrosion medium can further penetrate the interface between the substrate and the coating, then the corrosion occurred.^11,53^ Highly dispersed nano-Al_2_O_3_ particles in the epoxy matrix can provide a tortuous path to prevent H_2_O, O_2_ and Cl^−^ ions from penetrating through the coating (Fig. 14(b)).^54^ The enhancement of corrosion protection using the m-Al_2_O_3_/epoxy coating could be attributed to the reasons:^22^ (1) the epoxy resin could be regarded as a physical barrier coating, (2) Al_2_O_3_ had anti-corrosion performance inherently, (3) the nanoparticles provided an extra barrier layer to preeminently obstruct micropores for electrolyte permeation, which prevented the underlying metal from corrosion attack, and (4) the well-dispersed m-Al_2_O_3_ nanoparticles in epoxy resin prevented corrosion due to a relatively high aspect ratio, which could enhance corrosion resistance of the composite coatings. The mGO nanosheets were added and stacked in the epoxy matrix, which was used as an effective barrier to capillary pores diffusion, and to prolong the tortuosity of the diffusion pathway of O_2_ molecules to penetrate the coatings (Fig. 14(c)). Consequently, the addition of mGO could enhance corrosion resistance of the epoxy coating. In addition, because the few-layer mGO nanosheet has good electrical conductivity, the electrons generated by the oxidation reaction could be quickly migrated from the corrosion sites and thus the corrosion behavior of the coating would be accelerated. In this study, the abrasion and corrosion resistance of the mGO/epoxy coatings were less than those of the m-Al_2_O_3_/epoxy coatings. However, the corrosion resistance of mGO/epoxy composite coating was better than that of neat epoxy coating, according to the results of salt spray and electrochemical corrosion tests. On the one hand, the mGO nanosheets were agglomerated in the epoxy (see Fig. 7(e)–(g)). On the other hand, the mGO nanosheets were not horizontally distributed in the epoxy. Moreover, the corrosion resistance mechanism of m-Al_2_O_3_@GO/epoxy coating is shown in Fig. 14(d). In an ideal state, m-Al_2_O_3_@GO hybrids nanoparticles are horizontally distributed in the epoxy resin, which can not only improve the corrosion resistance, but also improve the hardness and impact wear resistance. However, in this study, the corrosion performance of m-Al_2_O_3_@GO/epoxy composite coating is not good, compared with the m-Al_2_O_3_/epoxy and mGO/epoxy coatings, which was mainly due to the combination of m-Al_2_O_3_ nanoparticles and flake mGO nanosheets. The hybrid ratio of GO and Al_2_O_3_ is also an important key factor, because the settling of Al_2_O_3_ nanoparticles on the graphene surface not only inhibited the electron transfer but also eliminated the agglomerations of graphene.^24^ The future work of this study is to find the optimal ratio between GO and Al_2_O_3_ to achieve the maximum possibility of synergy effect.

In this study, the GO content might be high, resulting in the agglomeration of GO nanosheets in the epoxy, indicating that the effect of mechanical stirring for the dispersion of GO nanosheets was not good. At the same time, the addition of small amount of Al_2_O_3_ could not isolate the graphene sheets. Uneven bonding of m-Al_2_O_3_ on GO nanosheet will lead to the fold, inclination and wave shape of mGO-Al_2_O_3_ hybrids (Fig. 14(d)), which is difficult to be evenly distributed in the epoxy resin horizontally, resulting in the bonding and folding among m-Al_2_O_3_@GO hybrids, and making the substrate contact with external corrosive media to form electrochemical corrosion, like a micro-battery, and further to accelerate the corrosion of the composite coating. To exploit the corrosion resistance of GO for the epoxy coating, several techniques have been reported to eliminate the direct intercontact of GO sheets and to retain the insulative epoxy composite coating. These techniques are based on modifying the GO with insulative ceramic nanoparticles.^55–57^ Therefore, the inclusion of these nanoparticles acted as intercalates between GO layers, which not only inhibited the electron transfer but also prevented the reagglomeration of GO. The solubility of GO in organic solvent was restricted by grafting organic functional groups to some extent, and the grafting reaction was complicated with poor efficiency.^58^ In this work, m-Al_2_O_3_ nanoparticles can't reduce the agglomeration of GO in the hybrids, and can't efficiency contribute to performance of GO in epoxy coating. This is attributed to the m-Al_2_O_3_@GO hybrids without modified GO sheets.

## 4 Conclusions

Nano-Al_2_O_3_ particles and graphene oxide (GO) nanosheets were successfully modified by KH550 and m-Al_2_O_3_@GO hybrids were synthesized using a facile approach. Then, the epoxy resin was filled with m-Al_2_O_3_, mGO and m-Al_2_O_3_@GO hybrids. The dispersibility and stability of particles in the epoxy resin had an influence on the hardness, abrasion and corrosion resistance of the composite coatings. The main conclusions were summarized below:

(1) With the addition of small amount of nanoparticles, the dispersion of nanoparticles in the epoxy resin was good. When the content of m-Al_2_O_3_ was equal to 1.5 wt%, the m-Al_2_O_3_ nanoparticles in the epoxy exhibited the best dispersibility and stability. However, the dispersibility and stability of mGO and m-Al_2_O_3_@GO nanofillers by mechanical stirring in the epoxy resin were not good.

(2) The hardness of the composite coatings increased with increasing particle content and decreased after exceeding a certain content of the particles, which was related to the dispersibility and stability of the particles in the epoxy resin.

(3) The m-Al_2_O_3_/epoxy composite coatings had better abrasion resistance, compared with the mGO/epoxy and m-Al_2_O_3_@GO/epoxy composite coatings with different degrees of the damaged area. The composite coatings incorporated with 1.5 wt% m-Al_2_O_3_, 0.2 wt% mGO and 1.0 wt% m-Al_2_O_3_@GO had relatively good abrasion resistance.

(4) For the different composite coatings, the coatings with less content of nanofillers showed relatively better corrosion resistance. And the corrosion resistance of the m-Al_2_O_3_/epoxy coatings was better than the mGO/epoxy and m-Al_2_O_3_@GO/epoxy coatings. The corrosion resistance of the composite coatings was sorted in the order of 1.5 wt% m-Al_2_O_3_/epoxy coating > 0.6 wt% m-Al_2_O_3_@GO/epoxy coating > 0.4 wt% mGO/epoxy coating > the neat epoxy coating.

## Conflicts of interest

There are no conflicts to declare.

## Supplementary Material

RA-012-D2RA04223A-s001
