# Supplementary material for: Incorporation of Al2O3, GO, and Al2O3@GO nanoparticles into water-borne epoxy coatings: abrasion and corrosion resistance
Source: RSC Adv. 2022 Aug 31;12(38):24804–20. doi: 10.1039/d2ra04223a (PMC9429057; doi:10.1039/d2ra04223a)
Supplement: RA-012-D2RA04223A-s001 [file RA-012-D2RA04223A-s001.pdf]

Electronic Supplementary Material (ESI) for RSC Advances.
This journal is © The Royal Society of Chemistry 2022

Incorporation of Al_2O_3, GO, Al_2O_3@GO nanoparticles into water-borne epoxy coatings: Abrasion and corrosion resistance

Jia-qi Huang[1], Kunming Liu[2], Xinlong Song[2], Guocheng Zheng[2], Qing Chen[3], Jiadi Sun[4], Haozhe Jin[5], Lanlan Jiang[1], Yusheng Jiang[1], Yi Zhang[1], Peng Jiang[1], Wangping Wu[1,*]

[1]*Electrochemistry and Corrosion Laboratory, School of Mechanical Engineering and Rail Transit, Changzhou University, Changzhou 213164, P.R. China*

[2]*Jiangsu Kexiang Anticorrosion Materials Co., Ltd, Changzhou 213100, China*

[3]*Zhejiang Fangyuan Test Group, Hangzhou 310018, China*

[4]*CNOOC Changzhou Paint and Coatings Industry Research Institute Co., Ltd., Changzhou, 213016, China*

[5]*Key Lab. of Fluid Transmission Technology of Zhejiang Province, Zhejiang, Sci-Tech University, Hangzhou, 310018, China*

[*]Corresponding author: wwp3.14@163.com; wuwping@cczu.edu.cn

Experimental

The thickness of the coating was measured with a handheld coating thickness meter (BGD 542/2). Sets of several cross-hatched scratches, with interscratch distance of 1mm and cross scratches at ~90°, were made on the deposits after drying. A 3M tape (610-1PK special test tape produced by the 3M company) was applied over the grid, placing the center of the tape over the grid and in the area of the grid smoothing into place by a finger. After holding on 90 seconds, the tape was removed by seizing the free end and pulled it off rapidly at 90°. The adhesion force between the layer and the substrate was assessed according to the cross-cut testing standard of ASTM-D3359-09. The cross-cut tests were repeated at least twice for evaluating the adhesion force between the substrate and the layer.

(a)

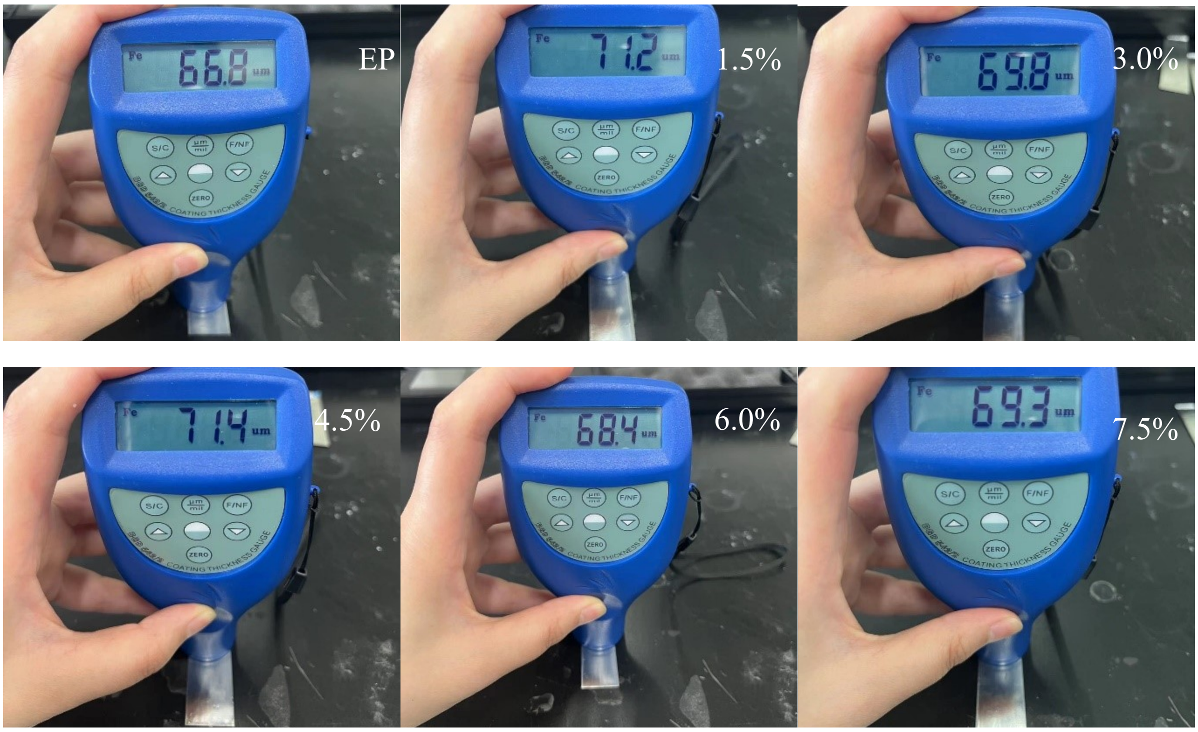
66.8 um
EP
71.2 um
1.5%
69.8 um
3.0%
71.4 um
4.5%
68.4 um
6.0%
69.3 um
7.5%

(b)

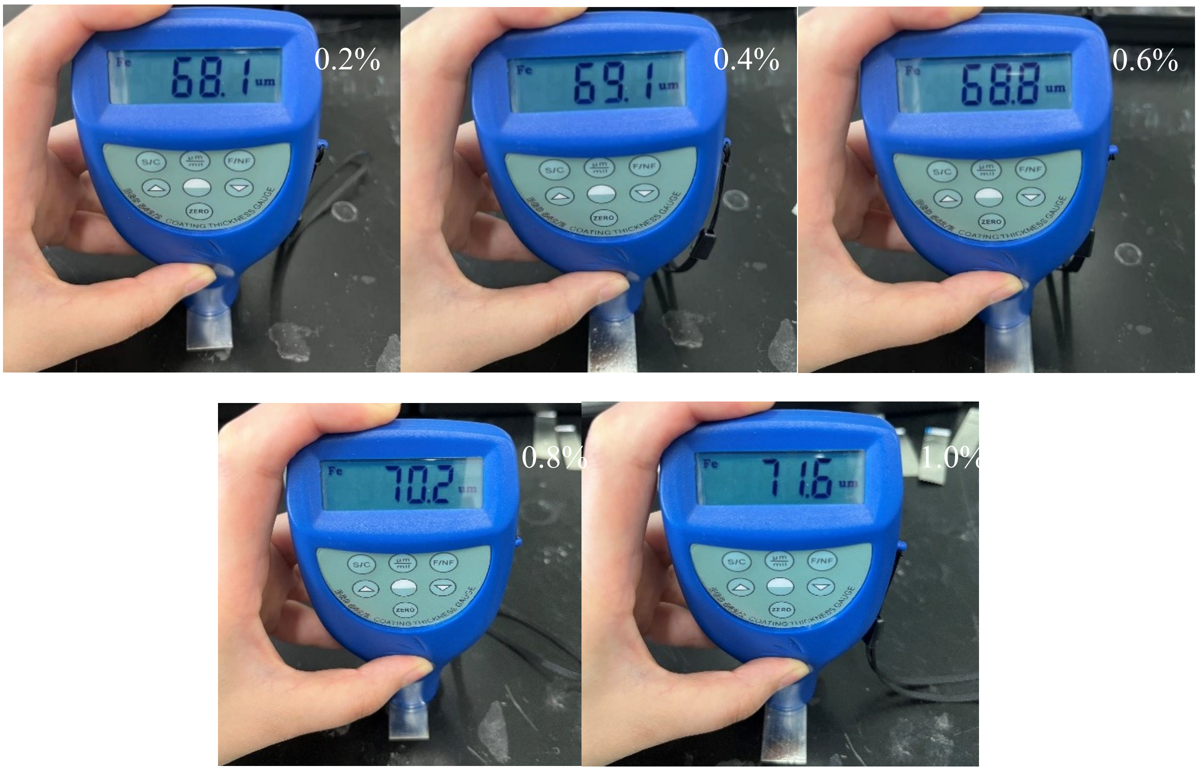
68.1 um
0.2%
69.1 um
0.4%
68.8 um
0.6%
70.2 um
0.8%
71.6 um
1.0%

(c)

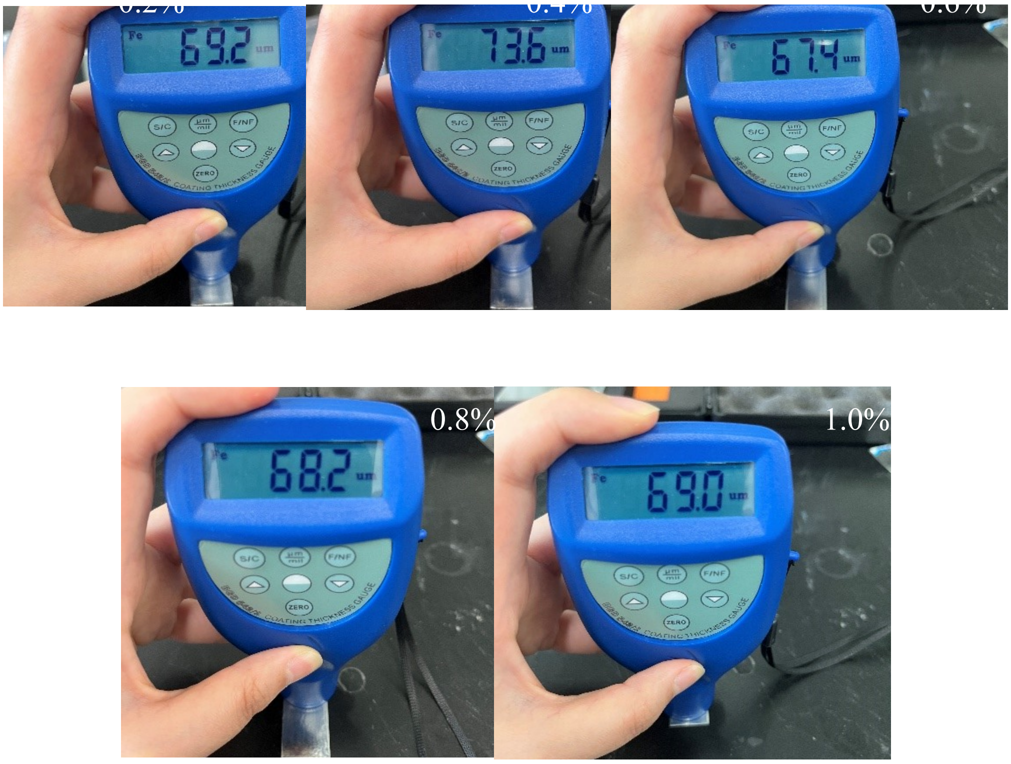

Figure 1S The thickness of the composite coatings, (a) the neat epoxy coating and m-Al_2O_3/epoxy coating, (b) mGO/epoxy coating and (c) m-Al_2O_3@GO/epoxy coating

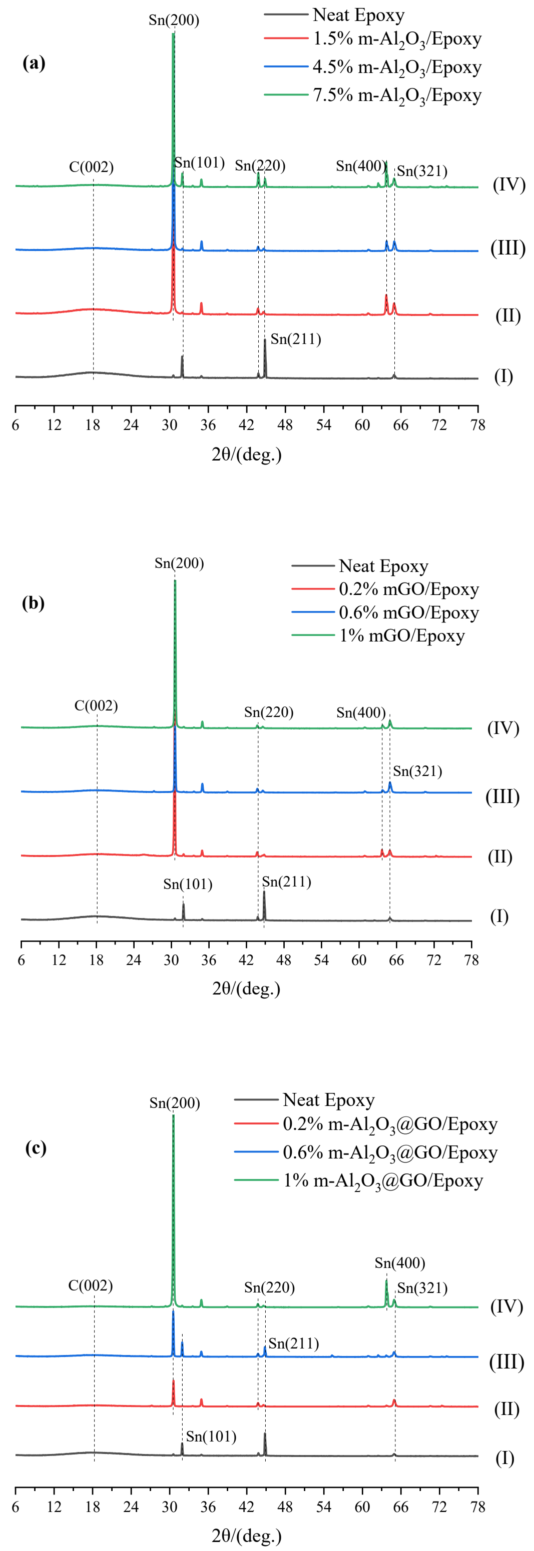

Figure 2S XRD patterns of (a) neat epoxy coating and m-Al_2O_3/epoxy coatings, (b) mGO/epoxy coatings and (c) m-Al_2O_3@GO/epoxy coatings

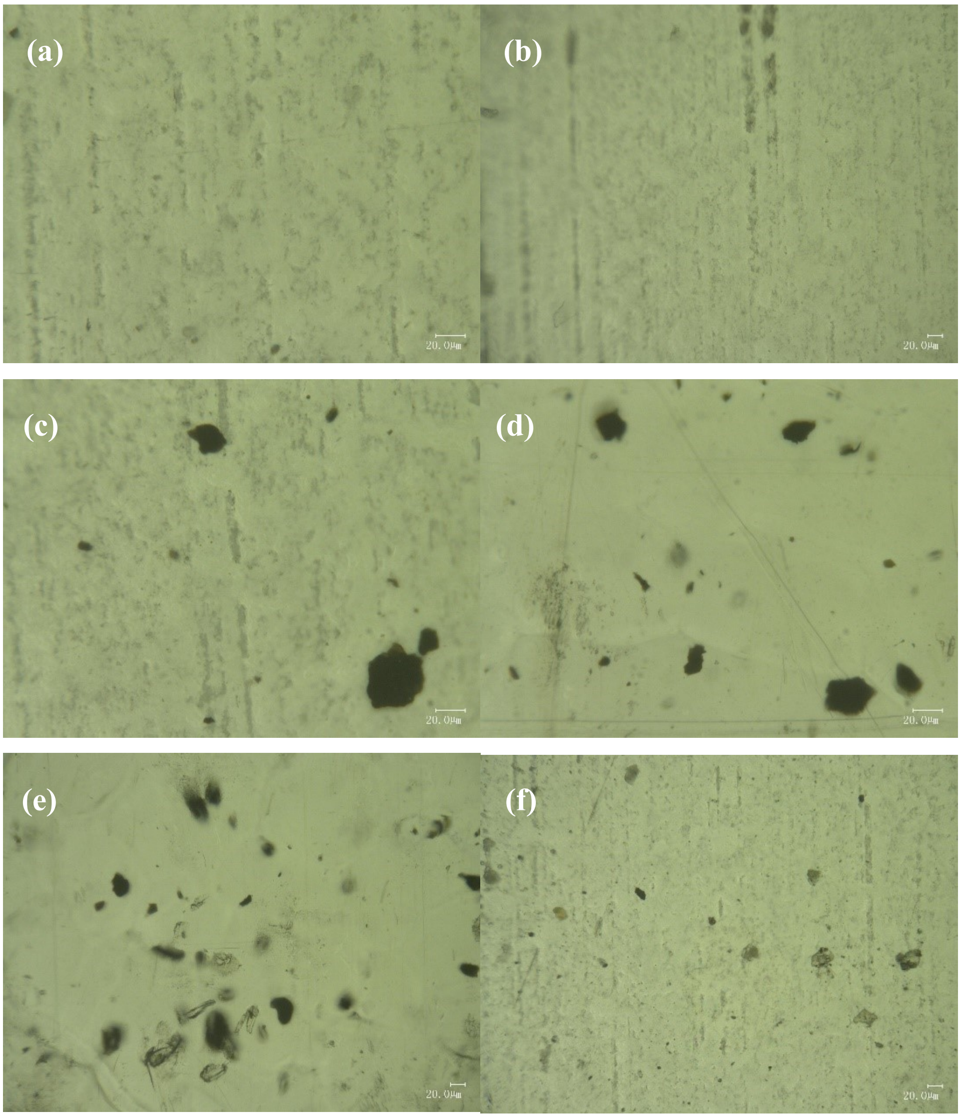

Figure 3S OM images of the composite coatings, (a) 3.0 wt% m-Al_2O_3/epoxy coating, (b) 6.0 wt% m-Al_2O_3/epoxy coating, (c) 0.4 wt% mGO/epoxy coating, (d) 0.8 wt% mGO/epoxy coating, (e) 0.4 wt% m-Al_2O_3@GO/epoxy coating, (f) 0.8 wt% m-Al_2O_3@GO/epoxy coating

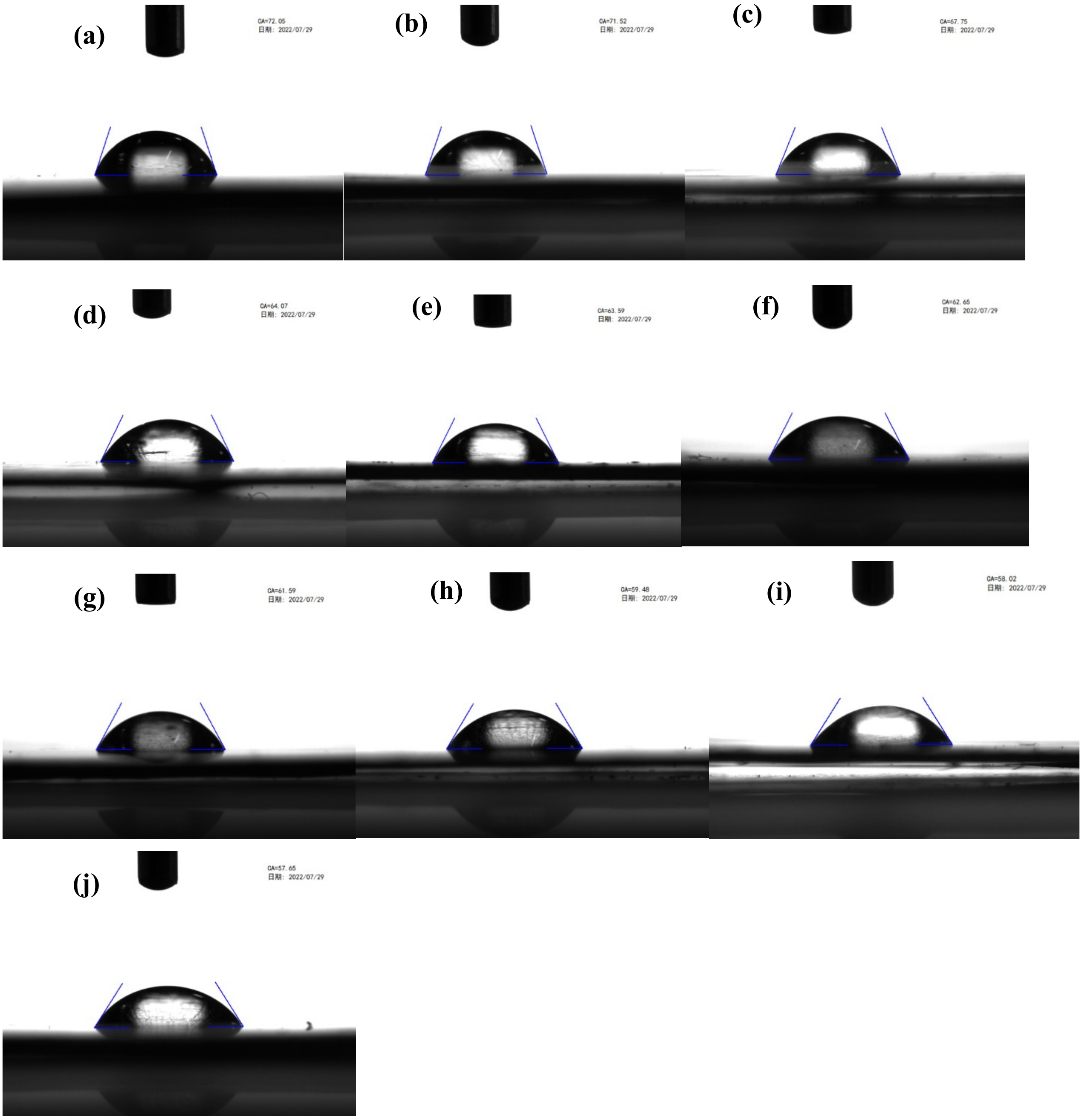

Figure 4S Contact angle of the coatings, (a) EP, (b) 1.5 wt% m-Al_2O_3/Epoxy, (c) 4.5 wt% m-Al_2O_3/Epoxy, (d) 7.5 wt% m-Al_2O_3/Epoxy, (e) 0.2 wt% mGO/Epoxy, (f) 0.6 wt% mGO/Epoxy, (g) 1.0 wt% mGO/Epoxy, (h) 0.2 wt% m-Al_2O_3@GO/Epoxy, (i) 0.4 wt% m-Al_2O_3@GO/Epoxy, (j) 1.0 wt% m-Al_2O_3@GO/Epoxy

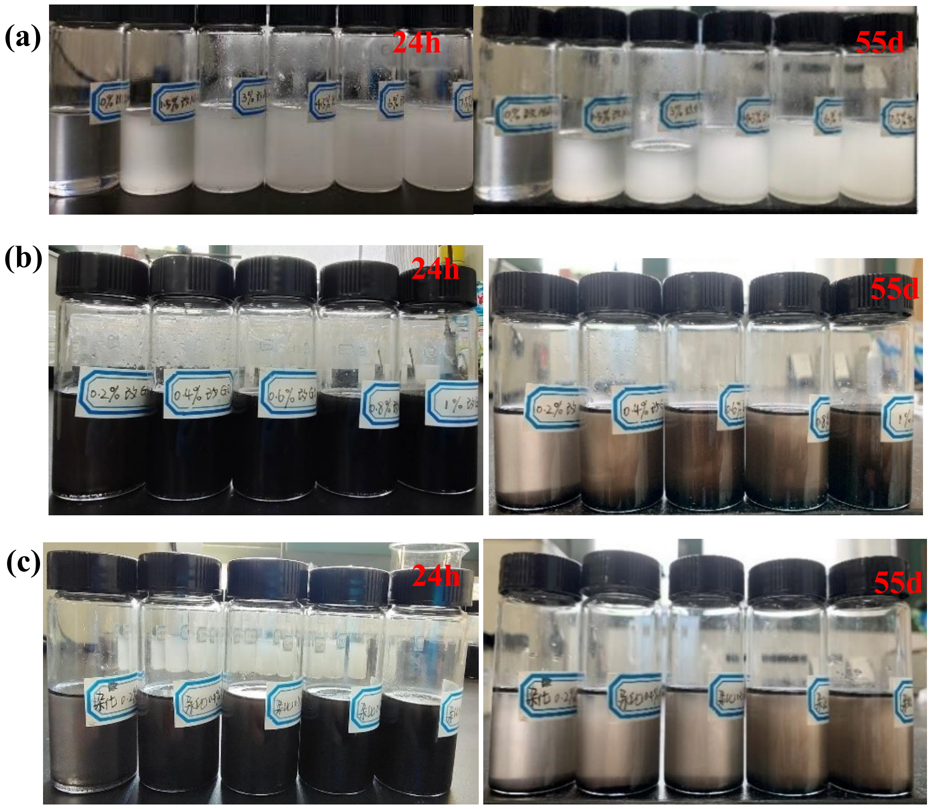

Figure 5S Dispersion and stability of nanoparticles with different contents in epoxy resin for different holding times, (a) m-Al_2O_3 (b) mGO and (c) m-Al_2O_3@GO hybrids

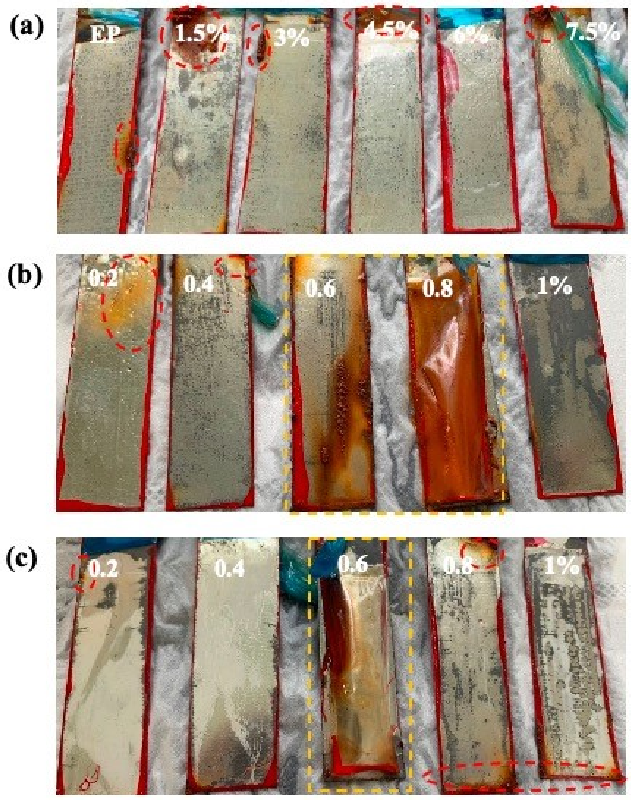

Figure 6S Digital images of the composite coatings immersed in 3.5 wt.% NaCl solution after 62 days (a) Neat epoxy and m-Al_2O_3/epoxy coatings, (b) mGO/epoxy coatings and (c) m-Al_2O_3@GO/epoxy coatings

Table 1S Evaluation of coating adhesion

Coating	Digital images of the coatings	Grade
Neat epoxy	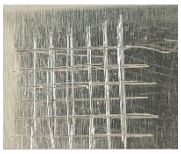	5B
1.5% m-Al_2O_3	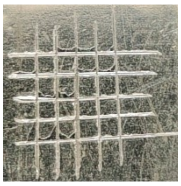	4B
3.0% m-Al_2O_3	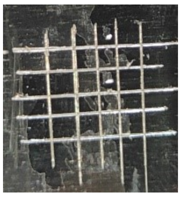	1B
4.5% m-Al_2O_3	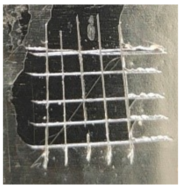	0B
6.0% m-Al_2O_3	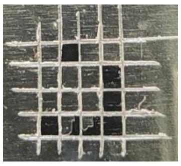	3B
7.5% m-Al_2O_3	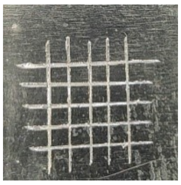	5B
0.2% mGO	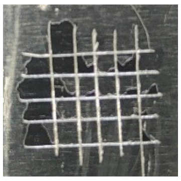	3B
0.4% mGO	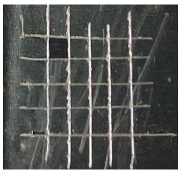	4B

0.6% mGO	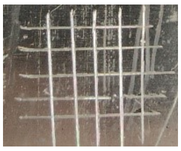	5B
0.8% mGO	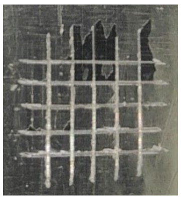	3B
1.0% mGO	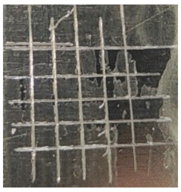	3B
0.2% m-Al_2O_3@GO	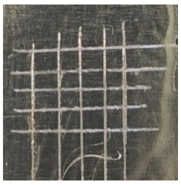	5B
0.4% m-Al_2O_3@GO	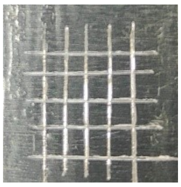	5B
0.6% m-Al_2O_3@GO	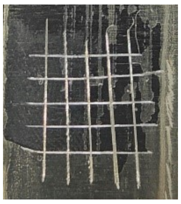	2B
0.8% m-Al_2O_3@GO	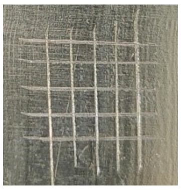	5B
1.0% m-Al_2O_3@GO	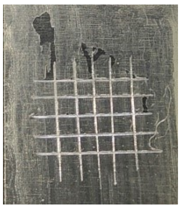	4B

The adhesive force between the coating and the substrate is evaluated by the tape peeling test. The epoxy resin coating is cut with a knife until it is cut to the substrate surface. The test results are shown in Table 1S. The binding force of the neat epoxy coating was 5B since there is no shedding phenomenon, the same as 7.5 wt% m-Al_2O_3/epoxy coating, 0.6 wt% mGO/epoxy coating and 0.2 wt%, 0.4 wt%, 0.8 wt% m-Al2O3@GO epoxy coating. However, the binding force of the coatings with the addition of particles was not significantly improved to some degree, because the agglomeration of nanoparticles influenced the adhesion of the interface between the coating and the smooth surface of the substrate. We did not focus on the adhesion of the coatings on the substrate, in this study. Of course, if we want to improve the adhesion, the surface of the substrate will be scratched by sandpapers, to make the surface rough, improving the adhesion of the coating.
